# Pathogen-Host Associations and Predicted Range Shifts of Human Monkeypox in Response to Climate Change in Central Africa

**DOI:** 10.1371/journal.pone.0066071

**Published:** 2013-07-31

**Authors:** Henri A. Thomassen, Trevon Fuller, Salvi Asefi-Najafabady, Julia A. G. Shiplacoff, Prime M. Mulembakani, Seth Blumberg, Sara C. Johnston, Neville K. Kisalu, Timothée L. Kinkela, Joseph N. Fair, Nathan D. Wolfe, Robert L. Shongo, Matthew LeBreton, Hermann Meyer, Linda L. Wright, Jean-Jacques Muyembe, Wolfgang Buermann, Emile Okitolonda, Lisa E. Hensley, James O. Lloyd-Smith, Thomas B. Smith, Anne W. Rimoin

**Affiliations:** 1 Center for Tropical Research, University of California Los Angeles, Los Angeles, California, United States of America; 2 Department of Comparative Zoology, University of Tübingen, Tübingen, Germany; 3 School of Life Sciences, Arizona State University, Tempe, Arizona, United States of America; 4 Institute of the Environment and Sustainability, University of California Los Angeles, Los Angeles, California, United States of America; 5 Kinshasa School of Public Health, Kinshasa, Democratic Republic of Congo; 6 Fogarty International Center, National Institutes of Health, Bethesda, Maryland, United States of America; 7 Department of Ecology and Evolutionary Biology, University of California Los Angeles, Los Angeles, California, United States of America; 8 United States Army Medical Research Institute of Infectious Diseases, Fredrick, Maryland, United States of America; 9 Department of Microbiology, Immunology, and Molecular Genetics, University of California Los Angeles, Los Angeles, California, United States of America; 10 Global Viral Forecasting, San Francisco, California, United States of America; 11 Stanford University, Program in Human Biology, Stanford, California, United States of America; 12 Ministry of Health, Kinshasa, Democratic Republic of Congo; 13 Bundeswehr Institute of Microbiology, Munich, Germany; 14 The Eunice Kennedy Shriver National Institute of Child Health and Human Development, Bethesda, Maryland, United States of America; 15 National Institute of Biomedical Research, Kinshasa, Democratic Republic of Congo; 16 Department of Atmospheric and Oceanic Sciences, University of California Los Angeles, Los Angeles, California, United States of America; 17 Medical Countermeasures Initiative, Silver Spring, Maryland, United States of America; 18 Department of Epidemiology, School of Public Health, University of California Los Angeles, Los Angeles, California, United States of America; Centers for Disease Control and Prevention, United States of America

## Abstract

Climate change is predicted to result in changes in the geographic ranges and local prevalence of infectious diseases, either through direct effects on the pathogen, or indirectly through range shifts in vector and reservoir species. To better understand the occurrence of monkeypox virus (MPXV), an emerging Orthopoxvirus in humans, under contemporary and future climate conditions, we used ecological niche modeling techniques in conjunction with climate and remote-sensing variables. We first created spatially explicit probability distributions of its candidate reservoir species in Africa's Congo Basin. Reservoir species distributions were subsequently used to model current and projected future distributions of human monkeypox (MPX). Results indicate that forest clearing and climate are significant driving factors of the transmission of MPX from wildlife to humans under current climate conditions. Models under contemporary climate conditions performed well, as indicated by high values for the area under the receiver operator curve (AUC), and tests on spatially randomly and non-randomly omitted test data. Future projections were made on IPCC 4^th^ Assessment climate change scenarios for 2050 and 2080, ranging from more conservative to more aggressive, and representing the potential variation within which range shifts can be expected to occur. Future projections showed range shifts into regions where MPX has not been recorded previously. Increased suitability for MPX was predicted in eastern Democratic Republic of Congo. Models developed here are useful for identifying areas where environmental conditions may become more suitable for human MPX; targeting candidate reservoir species for future screening efforts; and prioritizing regions for future MPX surveillance efforts.

## Introduction

Climate change is predicted to result in shifts in the incidence and prevalence of infectious diseases (e.g. [1], [2]), and may impose health risks on human populations in previously unexposed regions. As a result, the incidence of a variety of viruses is predicted to increase. Examples include arboviruses such as dengue, blue tongue, and African horse sickness viruses; tickborne encephalitis virus in the UK; and Usutu virus in Austria [3], [4], [5], [6], [7]. To date, climate-related studies of such diseases have focused on vector-borne pathogens (reviewed in [2]) with little attention paid to viruses with vertebrate reservoirs. The effect of climate on this type of viruses is likely to be complex since the host species themselves have niches limited by a variety of environmental conditions. Yet, a better understanding of the ecology of viruses and the ability to predict future outbreaks will be helpful in identifying potential risk areas for human infection, and in subsequent disease management and control efforts.

An emerging infectious disease of concern in tropical Africa is monkeypox (MPX). MPX virus (MPXV) is a zoonotic Orthopoxvirus that can cause serious smallpox-like illness in humans. It is endemic to forested regions of West and Central Africa, and since the global eradication of smallpox in 1977, MPXV has been considered the most important poxvirus affecting human health [8]. Once considered a rare, sporadic infection in humans, recent studies in the Democratic Republic of the Congo (DRC, previously Zaire), where most cases have been reported, suggest that the incidence of human MPX has markedly increased in the Congo Basin since the 1980s [9]. The post-eradication cessation of routine smallpox vaccination, which is known to provide cross-immunity against infection with MPXV, is likely to have played a role in the observed increase over the past three decades. Other driving factors such as land use and climate change likely contribute to the increase in incidence of MPX, but have so far not been studied.

The rise in incidence of MPX and its emergence in areas where it has not been previously known to occur [10] are important reminders that Africa is particularly vulnerable to emerging infectious diseases [1], [11]. First, Africa is the continent projected to be most severely impacted by climate change [12], likely affecting the distributions and prevalence of infectious diseases [1], [2]. Predictions for the end of the 21^st^ century are that annual mean temperatures will increase by up to 5°C, precipitation will significantly increase in tropical Africa and decrease further away from the equator, evaporation will increase across most of the continent, and seasonality in precipitation will be significantly affected (multi-model mean predictions from the IPCC 4^th^ Assessment Report [12]). Rainfall in West Africa is linked to sea surface temperatures in the Atlantic Ocean, and increasing temperatures could perturb the system leading to century-long droughts [13]. Second, human populations in Africa have a high disease burden [11], and may suffer from multiple infections and other health stressors such as malnutrition, which may depress immune response and increase susceptibility to new pathogens. In some populations of Sub-Saharan Africa, co-infection with one pathogen has been shown to increase the virulence of another [14]. These populations, which have a high incidence of HIV and other endemic infections, are potentially more sensitive to MPXV. Third, the health infrastructure in place is inadequate as a result of limited financial resources. These issues are further exacerbated by armed conflicts and the complexity of government institutions that have resulted in a large number of internally displaced persons (IDP), estimated to be around two million in 2009 [11], [15], [16]. Given these challenges, there is an urgent need to examine the effects of climate change on infectious diseases in Africa in order to most efficiently direct limited health resources [11].

Human MPX has been reported throughout most of tropical Africa, but the majority of cases are from the Congo basin (e.g. [23] and references therein). Little is known about the ecology of MPXV and its dependence on environmental conditions. The majority of human MPX infections result from close contact with infected animals that often serve as food sources, but person-to-person transmission occurs (e.g. [17]) and may be on the rise. Human-to-human transmission might spread monkeypox more widely as humans become more mobile, but third and fourth generation cases appear to be very rare, the transmission cycle is likely to cease relatively quickly, and the virus cannot sustain large-scale outbreaks in human populations in the rural settings where it has been observed [17]. . Antibodies to MPXV have been detected serologically in multiple animal species, suggesting that the host range could be quite large. A variety of potential reservoir species have been identified, including rope squirrels (genus *Funisciurus*; [18], [19], [20], [21]) and African dormice (genus *Graphiurus*; [21]). An outbreak in prairie dogs in the United States in 2003 occurred via imported rodents and caused clinical and subclinical cases in humans (e.g. [24]). A recent review of models for zoonotic infections highlighted MPXV as a key priority for modeling research [22].

**Figure 1:**
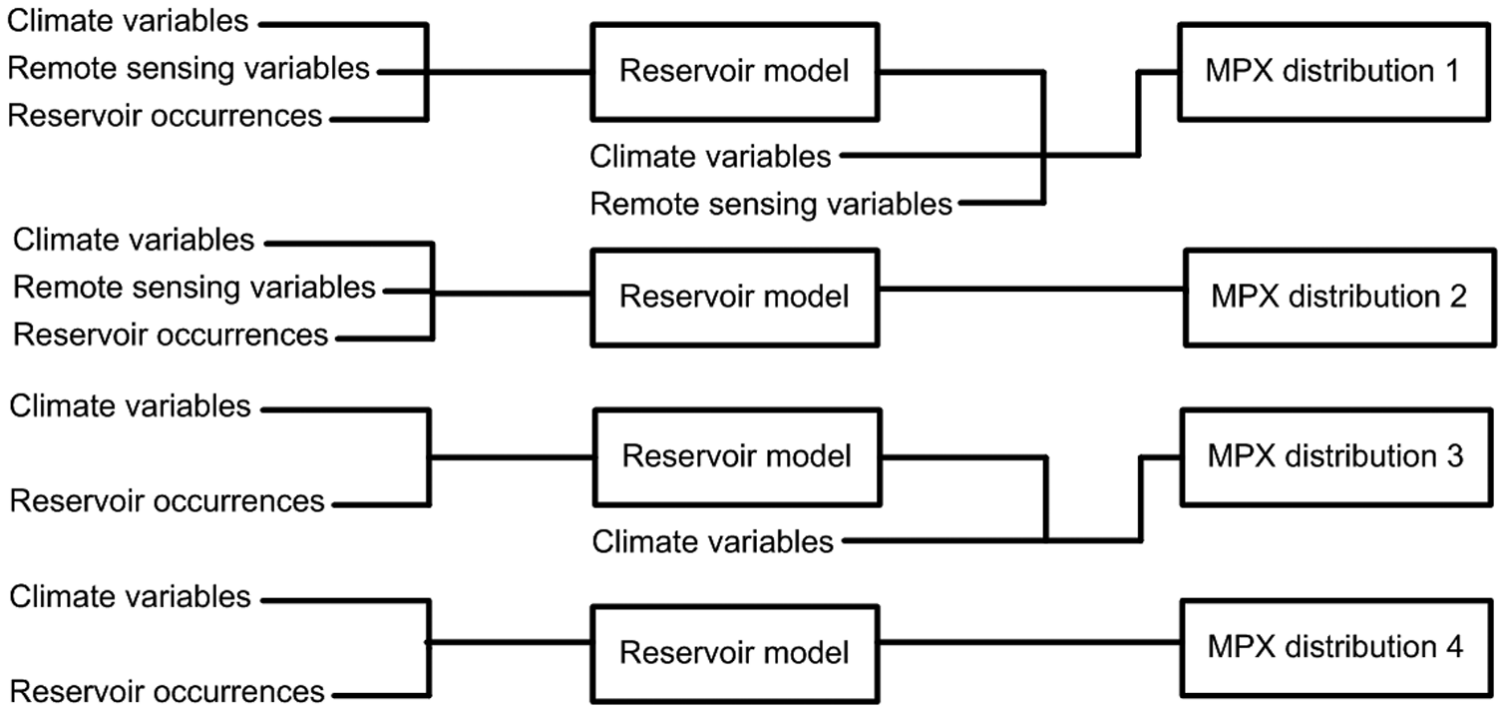
Flowchart of models created using the indicated input data. We modeled human MPX distributions under current conditions and assessed the concordance among models using the following predictor datasets: 1) reservoir species distributions (based on climate and remote sensing variables) and in addition the climate and remote sensing variables (the full model), which may contain additive information on top of the reservoir species distributions that were also based on both types of data; 2) only reservoir species distributions (based on climate and remote sensing variables); 3) reservoir species distributions (based on climate variables) plus climate variables; and 4) only reservoir species distributions (based on climate variables). To project future human MPX distributions under different climate change scenarios, we projected reservoir species distributions onto future climate variables. We then used the results as input for models of human MPX under approaches 3) and 4) above, since future remote sensing variables are not available.

New developments in spatial modeling and the availability of global climate data and satellite remotely-sensed variables characterizing the environment have provided the tools necessary for a better understanding of diseases' spatial ecology [25], [26], [27]. Few studies have predicted shifts in reservoir species and the infectious diseases associated with them, such as presented here, yet some recent studies have shown the potential of such an approach [28], [29]. Using these approaches, we recently demonstrated the importance of rope squirrels (*Funisciurus*) in predicting the locations of human MPX cases in the Sankuru district, DRC [30]. Here, we expand our study to a larger set of potential reservoir species, and to projections of human MPX occurrence under future climate conditions. We examine the spatial heterogeneity of human MPX across Tropical Africa, focusing on the Congo Basin. Our study focuses on both a small geographical scale combined with a short timeframe, as well as a large geographical scale combined with human MPX and environmental data covering multiple decades. To better understand the potential impacts of future climate change on the distribution of MPXV in Central Africa, we: 1) examine the relative contributions of climate and deforestation over the past decade to MPX occurrence in DRC (small scale, short timeframe); 2) identify the potential reservoir species that best predict human MPX occurrence (large scale, multi-decadal); 3) predict the distribution of MPXV across Tropical Africa under current climate conditions (large scale, multi-decadal); and 4) project the current distribution of MPXV onto future climate layers (large scale, multi-decadal). A schematic representation of aims 2), 3), and 4) is shown in Fig. 1. With the approach presented here, we aim to contribute to the development of a toolbox that is generally applicable to model a broad range of emerging infectious diseases with vertebrate reservoirs.

## Results

### Assessing the effects of present-day climate and forest clearing on MPX outbreaks on a local scale in DRC

**Figure 2:**
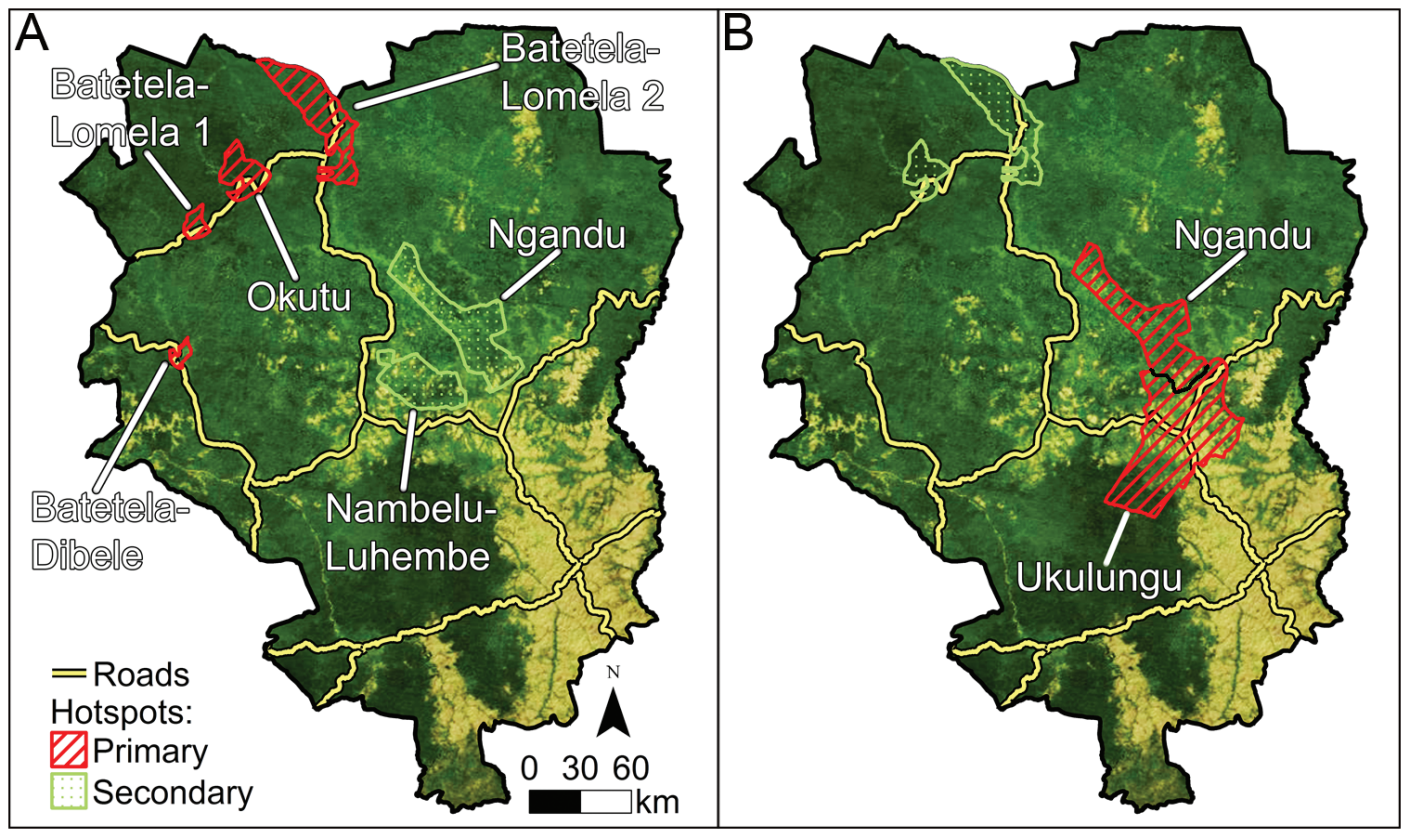
MPX hotspots in Sankuru district DRC. (A) secteurs classified as hotspots by a model that assumes that ecological factors are constant across the district; (B) MPX hotspots according to the model adjusted for spatial heterogeneity in deforestation and climate.

To test whether covariates such as deforestation since 2000 and climate affected the locations of MPX hotspots in Sankuru, DRC, as observed since 2001, we constructed a discrete Poisson model without covariates followed by a model that included ecological covariates and compared the hotspots indicated by the two models. The model without covariates identified clusters of MPX cases based only upon the fraction of the human population infected with MPX in each secteur. The ecological covariates were factors hypothesized to control habitat suitability for MPX reservoirs and included canopy moisture, a measure of drought, the deforestation rate, and the temperature of the coldest and the warmest quarter of the year (see Materials and Methods for details and Text S2 for a description of the drought calculation). If these ecological covariates were important drivers of MPX infections in humans, we would expect the model with covariates to classify different secteurs as MPX hotspots than the model without covariates. According to the model without covariates (Fig. 2A), the prime hotspots of MPX are in three secteurs in northwestern Sankuru: Batetela-Lomela, Okutu, and Batetela-Dibele. These secteurs are smaller than other secteurs in Sankuru (*t* = −4.712, *df* = 22.957, *p* = 4.79×10^−5^) but the fraction of the human population infected with MPX in these secteurs is significantly higher than average for Sankuru (*t* = 2.7289, *df* = 4, p = 0.0263). This preliminary model also identified a second hotspot in Ngandu in eastern Sankuru.

Next, we adjusted the preliminary model, which assumed that the ecological covariates were the same throughout Sankuru, to account for differences in rainfall, temperature, and the deforestation rate among the secteurs. Forest clearing emerged as an important driver of MPX transmission risk because including deforestation in the model reduced the log likelihood from 35.998 to 29.775, indicating that the deforestation-adjusted model provided a better fit to the data ([31] Table 1). Climate was also significant; adjusting the model to account for temperature and rainfall variation within Sankuru reduced the log likelihood to 12.44. Both the model with no covariates and the model adjusted for deforestation and climate identify Batetela-Lomela and Okutu in northwestern Sankuru and Ngandu in northeastern Sankuru as MPX hotspots (Fig. 2). However, according to the model with no covariates, Batetela-Lomela and Okutu are the primary hotspots and Ngandu is a secondary hotspot, whereas in the model adjusted for climate and deforestation, Ngandu becomes the primary hotspot. Furthermore, the climate- and deforestation-adjusted model identifies Ukulungu, a secteur south of Ngandu, as part of the primary MPX hotspot (Fig. 2B).

**Table 1:** Effect of recent forest clearing and climate on hotspots of MPX transmission in Sankuru, DRC.

Covariates	Hotspot	Secteurs	Observed No. MPX Cases	Expected No. MPX Cases	RR	LLR	*p*
None	P	Batetela-Lomela 1,2,3, Okutu, Batetela-Dibele	33	5.07	7.6	35.998	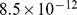
	S	Nabelu-Luhembe, Ngandu1	45	17.7	2.99	16.823	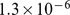
Deforestation	P	Ngandu1,2 Watambulu-Sud BahambaII, Ukulungu Watambulu Batetela-Lomela3, Nambelu-Luhembe	121	67.4	3	29.775	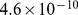
	S	Batetela-Dibele Batetela-Lomela1	15	0.82	19.61	29.8756	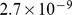
Climate*	P	Ukulungu, Ngandu1	34	13.3	2.87	12.4	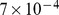
	S	Batetela-Lomela2,3, Okutu	18	5.85	3.28	8.46	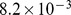
Deforestation+Climate	P	Ukulungu, Ngandu1	34	13.68	2.79	11.77	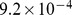
	S	Batetela-Lomela3, Okutu	17	4.85	3.73	9.55	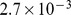

* The climate variables were temperature of the warmest quarter, temperature of the coldest quarter, canopy moisture, and the correlation between maximum water deficit, which is a measure of drought, and the Atlantic Multidecadal Oscillation (for details, see Table 6). P = primary hotspot, *p* = *p*-value, LLR = log likelihood ratio, RR = relative risk, and S = secondary hotspot.

### Regional scale study of MPX under current climate conditions

We used ecological niche modeling to first reconstruct the potential distributions of reservoir species, which were used in subsequent models of human MPX occurrence. Models for reservoir species based on climate variables performed well, as suggested by AUC values >0.85 and test AUC values >0.80 (Table 2), and by model tests using spatial subdivision of the presence points into training and test data sets (Table 3). The only model that did not perform significantly better than random was the one for the fire-footed rope squirrel (*F. pyrropus*) using western occurrence data to train the model. This may be explained by a relative lack of occurrence data in Central Africa (Nigeria, Cameroon), combined with a relatively high density of occurrences in West Africa (Ivory Coast, Ghana). Model tests using a spatial separation of eastern and western sites were qualitatively the same when the “minimum training presence” threshold was employed compared to those based on the “balance” threshold (see Materials and Methods). Temperature seasonality and annual precipitation stood out as important variables in predicting reservoir species' distributions (Fig. S1). The predicted distributions were largely confined to the African tropical forests (Fig. S2), and corresponded well with known distributions based on expert knowledge [33], [34]. The modeled distributions were therefore considered useful for subsequent application as predictor variables in modeling human MPX.

**Table 2:** Results of Maxent runs for reservoir species.

Species common name	Species scientific name	N sites	AUC	Test AUC
African brush-tailed porcupine	*Atherurus africanus*	52	0.958	0.943
Long-tailed pangolin	*Manis tetradactyla*	100	0.893	0.810
Tree pangolin	*Manis tricuspis*	100	0.919	0.846
Demidoff's galago	*Galagoides demidoff*	100	0.923	0.855
Greater cane rat	*Thryonomys swinderianus*	65	0.888	0.858
Gambian rat	*Cricetomys gambianus*	127	0.911	0.870
Wolf's monkey	*Cercopithecus wolfi*	100	0.930	0.852
Grey-cheeked mangabey	*Lophocebus albigena*	100	0.923	0.865
Thomas's rope squirrel	*Funisciurus anerythrus*	75	0.951	0.937
Congo rope squirrel	*Funisciurus congicus*	48	0.938	0.930
Fire-footed rope squirrel	*Funisciurus pyrropus*	94	0.956	0.915

**Table 3:** Results of model performance tests for potential reservoir species using spatial subdivisions.

Species name	AUC		% area>threshold		P binomial test	
	W	E	W	E	W>E	E>W
African brush-tailed porcupine (*A. africanus*)	0.988	0.976	0.157	0.130	**<0.001**	**<0.001**
Long-tailed pangolin (*M. tetradactyla*)	0.944	0.925	0.252	0.370	**<0.001**	**<0.001**
Tree pangolin (*M. tricuspis*)	0.944	0.898	0.278	0.594	**<0.001**	**<0.001**
Demidoff's galago (*G. demidoff*)	0.943	0.902	0.274	0.466	**<0.001**	**<0.001**
Greater cane rat (*T. swinderianus*)	0.938	0.919	0.499	0.484	**0.003**	**0.030**
Gambian rat (*C. gambianus*)	0.936	0.969	0.370	0.234	**<0.001**	**<0.001**
Wolf's monkey (*C. wolfi*)	0.949	0.908	0.242	0.479	**<0.001**	**<0.001**
Grey-cheeked mangabey (*L. albigena*)	0.951	0.900	0.261	0.511	**<0.001**	**<0.001**
Thomas's rope squirrel (*F. anerythrus*)	0.947	0.978	0.278	0.113	**<0.001**	**<0.001**
Congo rope squirrel (*F. congicus*)	0.968	0.946	0.331	0.196	**<0.001**	**0.003**
Fire-footed rope squirrel (*F. pyrropus*)	0.969	0.978	0.295	0.225	0.374	**<0.001**

Shown are AUC values for models using points from the west (W) or the east (E); the percentage of the total study area predicted to be over the balance threshold; and the p-values for one-tailed binomial tests between the number of test points predicted to be suitable for the species and the fractional predicted area over the balance threshold. P-values in bold indicate models that performed significantly better than random; that is, significantly more test points were predicted to be suitable for the species than expected based on the fraction of the total area predicted to be suitable. W>E = western points were used for training, eastern for testing; E>W = eastern points were used for training, and western for testing.

**Figure 3:**
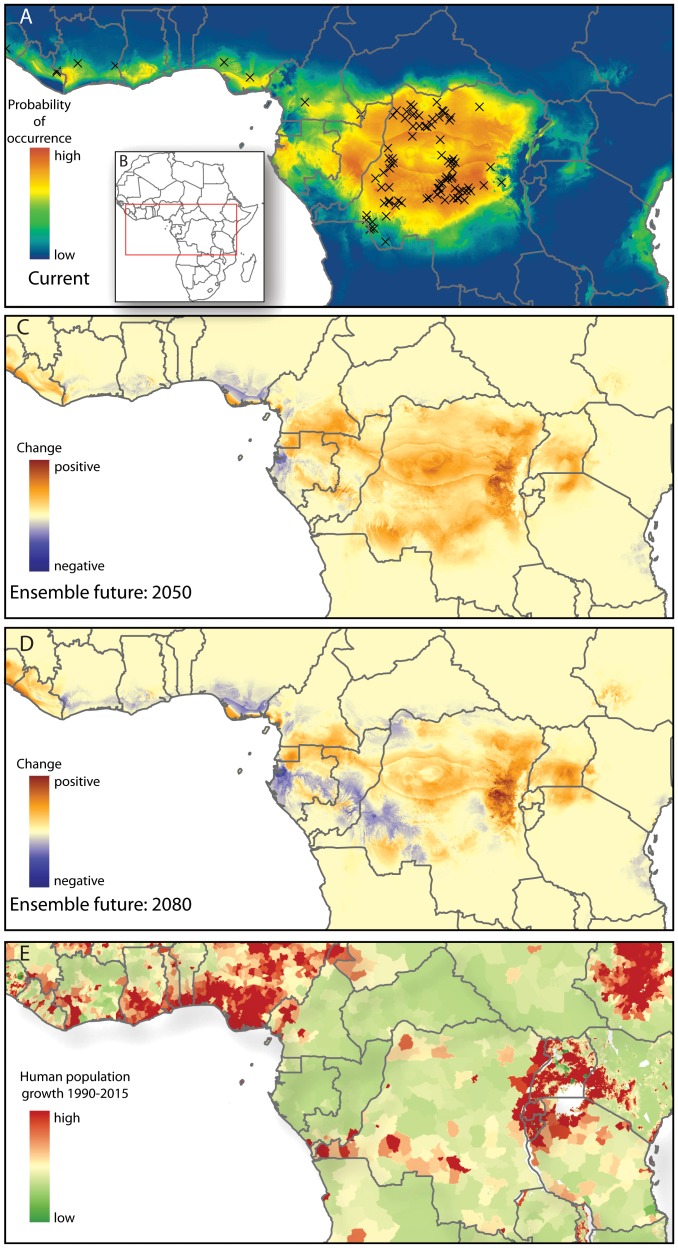
Observed and predicted human MPX occurrence. (A) Maxent prediction of human MPX occurrence under contemporary climate conditions, using reservoir species as predictor variables, with only linear ‘features’ (i.e. only linear coefficients are used for each predictor) allowed in the model. Colors indicate the probability of MPX occurrence, with cooler colors indicating lower probabilities and warmer colors higher probabilities (see color bar). Crosses indicate the reduced set of observed cases of MPX in humans (see Materials and Methods). (B) Study area. (C) Average projected change in probability of human MPX occurrence for eight climate change scenarios for 2050. (D) Average projected change in probability of human MPX occurrence under eight climate change scenarios for 2080. Colors in (C) and (D) indicate the change in probability of occurrence, with cooler colors indicating a decrease, and warmer colors an increase. (E) Projected human population growth from 1990–2015. The growth of the human population in the eastern DRC and Uganda, which borders North Kivu province in the eastern DRC, will be among the greatest of any region in central Africa. For areas shown in red, there has been a large increase in the human population since 1990 and further growth is forecast in the imminent future. Projections for later in the 21 century are qualitatively similar. For example, the population density of Uganda is forecast to increase 171% by 2050 [56]. A potential epicenter of future MPX outbreaks in the eastern DRC could arise from the confluence of population growth and increased MPX prevalence under climate change.

**Figure 4:**
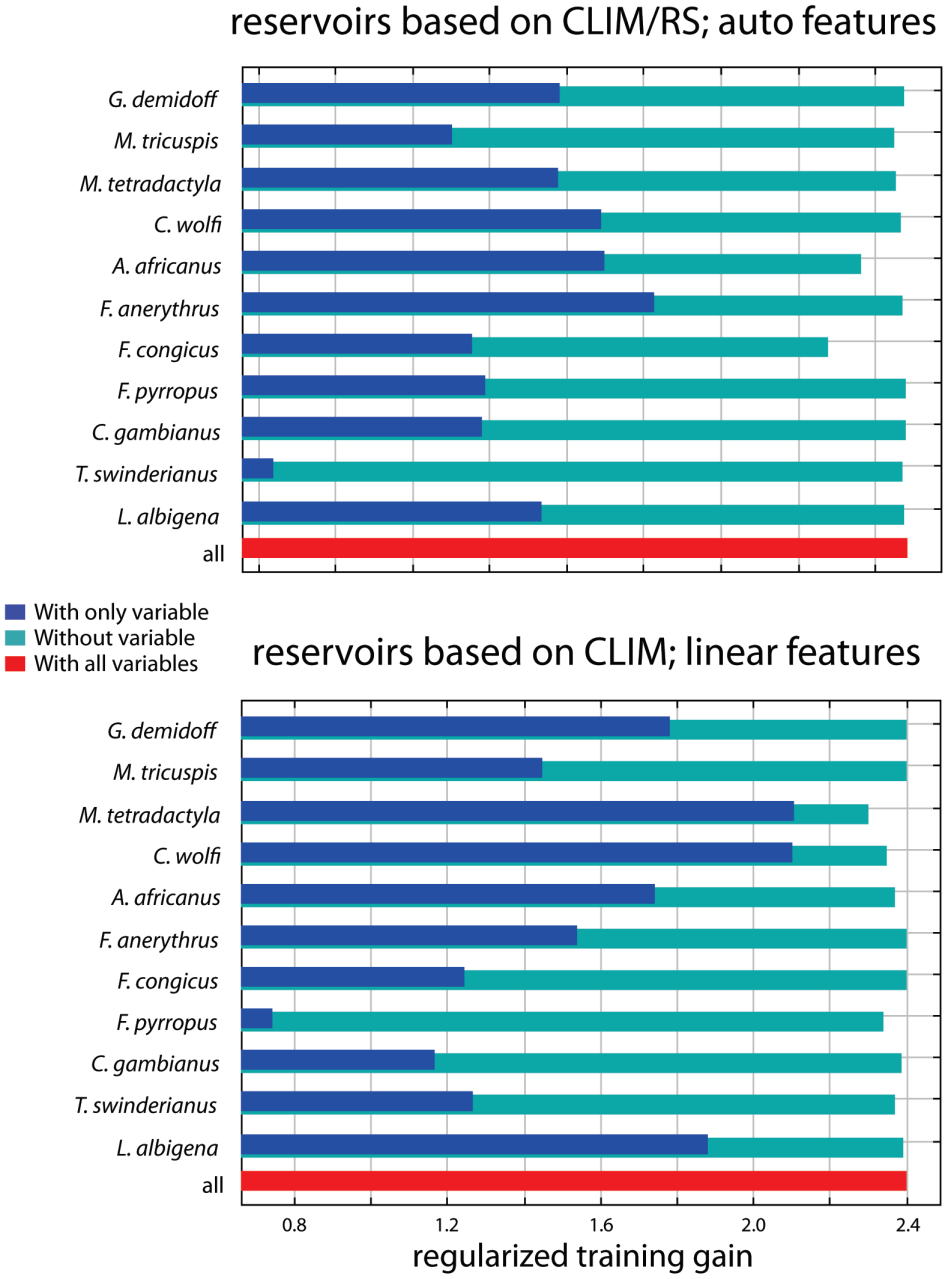
Variable importance of Maxent models of MPXV occurrence. Results are shown for a model that included reservoir species based on climate and remote sensing variables with all ‘features’ allowed (auto features, i.e. linear and quadratic coefficients can be used for each predictor, as well as step functions and interactions) (top panel), and a model that included only the reservoir species, based on climate variables, and with only linear ‘features’ (i.e. only linear coefficients are used for each predictor) allowed in the model (bottom panel). Dark blue bars indicate test results in which only the variable in question was entered into the model, and light blue bars in which all variables except the one in question were entered. Longer dark blue bars and shorter light blue bars indicate higher variable importance.

Results for models of human MPX under contemporary climate conditions are shown in Table 4. We assessed model accuracy using the Akaike Information Criterion with a correction for small samples sizes (hereafter “AICc”). The model with the minimum AICc was considered to have the best support. The lowest AICc value (2777.3), and thus the least complex model with high performance, was found when using reservoir species distributions as predictor variables, with only linear features allowed to be included in the model. The fact that a model with ‘auto features’ did not perform significantly better than a model with only linear features may be somewhat surprising given the often complex relationships between species' occurrence and environmental variables. In our case, however, this complexity of biotic and abiotic interactions may be sufficiently captured by the putative reservoir species' distributions, and result in relatively simple, monotonic relationships between reservoir and human MPX (note that features are similar to basis functions, and linear features can thus result in non-linear, monotonic response curves; see also [35]). Human MPX was predicted to be present in large parts of Central and Western Africa (Fig. 3A). In a model using reservoir species distributions that were based on both climate and remote sensing variables, Thomas's rope squirrel (*F. anerythrus*) was the most important variable when used on its own (Fig. 4). In the same model, the Congo rope squirrel (*F. congicus*) was the most important when omitted from the model, indicating that it contained unique information not captured by other reservoir species distributions. Variable importance changed when the reservoir species distributions were based on only climate data. In this case, rope squirrels were not the most important species, and instead the pangolin *Manis tetradactyla* was most important, both used on its own, or when omitted from the model. A close second in determining human MPX distribution in this model was Wolf's monkey (*Cercopithecus wolfi*). This difference in variable importance between the two models can likely be explained by the more fine-scale resolution of the remote sensing variables used in the former model as compared to the climate data used in the latter. The fine resolution may more effectively capture small-scale heterogeneity in human MPX occurrence that is not picked up by lower resolution variables. Nevertheless, the broader-scale predictions of human MPX occurrence were highly concordant among the different data sets used (Fig. S3), despite the fact that several reservoir models were more complex due to interactions among predictor variables. We therefore used the least complex model – which used reservoir species based on climate, and allowed for linear features only – in subsequent projections of human MPX occurrence from future reservoir species distributions.

**Table 4:** Results of Maxent runs for human MPX.

Predictor variable set	N sites	AUC	Test AUC	AICc
Res(CLIM/RS)+CLIM+RS	93	0.983	0.973	2924.6
Res(CLIM/RS)	93	0.989	0.974	2793.9
Res(CLIM)+CLIM	93	0.977	0.974	2784.9
Res(CLIM)	93	0.976	0.972	2797.2
Res(CLIM) – linear features	93	0.969	0.969	**2777.3**

Results are shown for models with different sets of input predictor variables. CLIM = climate variables; RS = remote sensing variables; Res() indicate reservoir species based on the variables in brackets; linear features = only linear features allowed in the Maxent model.

### Tests of human MPX model performance

We subdivided our study region into western (n = 45) and eastern (n = 48) sites. AUC values; balance thresholds, which balances the training omission rate, cumulative threshold, and fractional predicted area; and proportion of total area above the balance threshold for each of the models are shown in Table 5. Tests of model performance presented here are particularly conservative, because pseudo-absence background points were drawn from the entire study region, including the area where MPX positive locations were omitted (i.e. the western or eastern part of our study region). The threshold-dependent testing approach suggested that models trained on western sites predicted MPXV occurrence in their corresponding eastern test sites and vice versa significantly better than random (MPXV was predicted to be present in 31 out of 45 western sites when eastern sites were used as training data, and in 48 out of 48 eastern sites when western sites were used as training; one-tailed binomial tests, p<0.001).

**Table 5:** Results of model performance tests using spatial subdivisions.

Training set	Test set	N sites	AUC	Balance threshold	% area>balance threshold	P binomial test*	P Wilcoxon test*
West	East	45	0.960	0.048	0.183	**<0.001**	0.889
East	West	48	0.986	0.017	0.071	**<0.001**	**<0.001**

* The binomial test compares model performance to random performance, whereas the Wilcoxon signed rank test compares the predicted and observed logistic probabilities. Thus, good model performance is indicated by significant test results in the case of the binomial test, but non-significant results in the case of the Wilcoxon signed rank tests.

In addition, the threshold-independent test also suggested that a model based on western sites performed well in predicting suitability in eastern sites (1-tailed Wilcoxon signed rank test between models using western versus eastern sites as training sites: Z = 120.0, p = 0.889, suggesting there is no difference in predicted suitability of eastern sites between models using eastern or western sites as training data). However, eastern sites did not perform well in predicting the suitability for sites in the west (1-tailed Wilcoxon signed rank test between models using eastern versus western sites as training sites: Z = 500.5, p = 0.0001). This result may be attributed to the fact that the western sites comprise those from Central Africa, but the eastern sites do not include any from West Africa, where environmental conditions are rather different. Thus, the western sites span much of the environmental heterogeneity observed in the eastern sites, but the reverse is not true.

### Future projections

To project the distribution of human MPX under future climate conditions, we first projected reservoir species distributions on climate variables from the eight climate change scenarios each for 2050 and 2080. Changes in reservoir species distributions varied between decreasing or increasing suitability and in some cases showed considerable shifts in the geographic range (Fig. S2). For instance, the size of the distribution of the tree pangolin *M. tricuspis* was projected to decrease. In contrast, changing climate was projected to result in drastic range expansions of the rope squirrel *F. pyrropus*, whereas the African brush-tailed porcupine (*A. africanus*) was projected to retain nearly the same distribution. Very little to no clamping was detected, suggesting that the projected future environmental conditions in our study region were not more extreme than any observed currently. Subsequent projections for human MPX occurrence (based on projections of current reservoir species distributions on future climate conditions) showed strong patterns of change, where some regions were projected to become less suitable, and others to become more suitable (Fig. 3c, d for multi-model ensemble change maps and Fig. S4 for projections on each climate change scenario). Interestingly, the future projections suggest an overall increase in the geographic range of human MPX for 2050–2060, whereas a subsequent decrease is projected for areas in the southwest by 2080–2090. More specifically, by the turn of the century, human MPX is projected to become less common in much of western Africa, southern Gabon, central Republic of Congo, and parts of DRC. In contrast, increased suitability may result in a range shift eastwards in DRC and into Uganda, southwestern Kenya, and northwestern Tanzania. This shift is consistently predicted for all climate change scenarios (Fig. S4). In addition, southeastern Cameroon, northern Gabon, and Equatorial Guinea are projected to become much more suitable for human MPX than under current climate conditions.

The teardrop shape in central DRC (Fig. 3c–d) is a result of high precipitation in the driest quarter (Bio17) and potentially the bull's eye effect, an artifact of the interpolation method used in the WorldClim dataset in combination with relatively sparse climate station network in the area (1–25 stations per 0.5° grid cell, which is approximately 2500 km^2^); yet the sharp transitions are caused by the interaction of Bio17 and other climate variables.

Like WorldClim, the CRU climate data set, which is constructed from ground-based weather stations, also predicts that central DRC is currently wetter than western or eastern DRC [36]. However, satellite observations suggest that central DRC is drier than the west or east, which could be due to the divergence of air near the sides of the Congo River leading to low precipitation near the river [37]. Although future field work and regional atmospheric models are needed to validate the WorldClim rainfall estimates utilized here, the use of this data appears to be justified to the extent that it represents the best ground-based precipitation estimates currently available for the DRC.

## Discussion

Three-quarters of the emerging pathogens in humans are zoonotic in origin [38]. Thus, there is an urgent need to elucidate the geographic distributions of wildlife diseases. Our present analysis is a contribution toward the development of a methodology that can be used to predict the distribution of any virus with a wild mammal reservoir. This approach can potentially be adapted to a broad range of other emerging pathogens with mammalian reservoirs including hantavirus and Lassa virus [16], [39]. Furthermore, the approach reported here can be utilized to design inventories to identify the most important reservoirs for a range of pathogens in tropical forests. In particular, the distribution modeling techniques developed here can focus surveillance to a subset of species from an initial large set of potential reservoir species.

### Assessing the effects of present-day climate and forest clearing on MPX outbreaks in DRC

**Figure 5:**
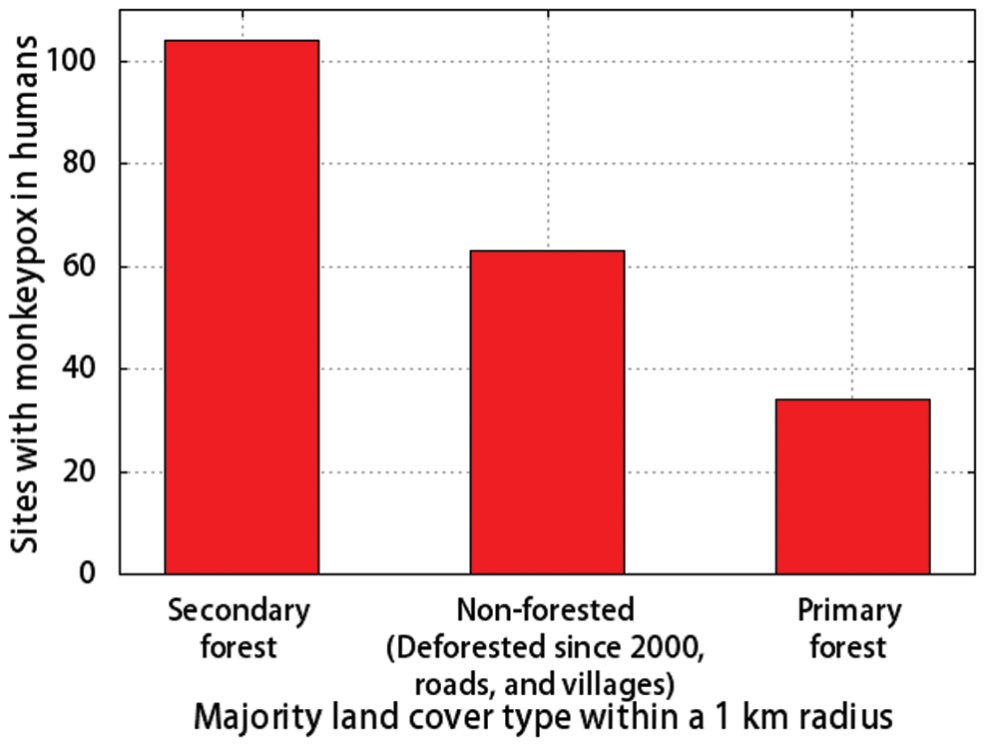
Land cover at sites with MPX infections in humans in Sankuru (2005–07). The land cover data are from [58].

Our local-scale study in Sankuru supports the hypothesis that climate and deforestation are both important drivers of MPX insofar as the model that included covariates related to climate and forest clearing provided the best fit to the data. Here we hypothesize about two mechanisms by which forest clearing and climatic factors might affect the transmission of MPXV from wildlife to humans. First, the clearing of primary forest by humans could increase habitats that are optimal for wildlife species that carry the virus, leading to higher abundances of these species, more frequent contact between humans and wildlife carriers, and increased transmission of the virus to humans. We note that increased habitat for wildlife hosts due to the clearing of primary forests was implicated as one of the main drivers behind the range expansion of Japanese Encephalitis Virus in Asia in the mid-1990s [40]. In Sankuru, the conversion of primary forest to agricultural fields could increase the habitat for MPX hosts such as rope squirrels, which inhabit secondary forest and ecotones between agricultural fields and forests [19], [20]. According to this hypothesis, human MPX infections should occur more often near secondary forests and recently deforested areas than near intact primary forest. Furthermore, we might expect that people who spend time in land that has recently been deforested or in land that represents a transition zone between forest and agricultural land would be infected with MPXV at a higher rate than other members of the population. The data on MPX cases in humans in Sankuru support this hypothesis to the extent that 83% of infections in humans were at sites surrounded either by secondary forest or formerly forested land that has been cleared in the past decade (Fig. 5, Fig. S5). In addition, boys, who typically play in agricultural areas along the perimeter of secondary forests, where they trap and eat small mammals, have the highest incidence of MPX in Sankuru [9].

Second, climatic factors could lead to increased MPX infections in humans in Sankuru because drought in forested areas could compel sylvan mammals that carry MPX to disperse into non-forested areas such as human settlements to forage. During these foraging bouts, small mammals infected with MPX could transmit the virus to villagers. If this hypothesis were correct, we would expect there to be more cases of MPX in secteurs where there has been severe drought in forested areas in the past decade. The data from Sankuru tend to support this hypothesis to the extent that Ngandu and Ukulungu, which emerged as the primary MPX hotspot, have had high water deficits in forested areas over the past decade, based on satellite observations (Fig. 2b, Text S1; Fig. S6). However, these results must be interpreted cautiously because additional ground-truthing is needed to confirm that water deficits estimated using remote sensing provide accurate estimates of canopy moisture in central DRC. At the national scale, there is a west-to-east gradient within DRC with respect to canopy water deficits measured by satellite. In western DRC, there is a significant negative correlation between canopy water deficits and the Atlantic Multidecadal Oscillation (AMO), which is a circulation pattern in the Atlantic Ocean that is correlated with climate change [41], [42]. Western DRC has a distinct strain of MPXV, whereas in eastern DRC, where there is no association between climate change and canopy water deficits estimated via remote sensing, a different MPXV strain is prevalent (Fig. S7). This could indicate that different species are the primary reservoirs in different habitat types. The data presented here are suggestive of a possible relationship between forest canopy moisture, climate change, and MPX, which merits further investigation.

Finally, we note that the two mechanisms considered here, drought and deforestation, may not be exclusive. A hypothesis that awaits confirmation via future field work is that drought could trigger desiccation and tree dieback in primary forests in DRC and that this might increase secondary forest that is optimal habitat for wildlife that carry MPXV. Although drought has been shown to trigger tree die-offs in the Amazon [43], additional field campaigns are needed to test whether the conversion of primary to secondary forest is tied to drought in the Congo Basin. An alternative hypothesis is that DRC's forests may have evolved resistance to water deficits because multidecadal droughts have occurred frequently in sub-Saharan Africa during the Holocene period [13]. If our preliminary data that are suggestive of a relationship between present day climate and MPX can be confirmed, this will provide further impetus for predicting how climate change in the coming decades will trigger increases in MPX infections in humans. The development of high resolution maps of future forest cover in Africa remains an important area for future research. However, since future climate maps are already available for Central Africa [44], we developed forecasts of the distribution of MPX under a range of climate scenarios for the 2050s and 2080s.

### Assessing the role of present and future climate change on MPX outbreaks at the regional scale in Tropical Africa

Our study uses the most extensive incidence data set of MPX occurrence yet developed, and is novel in using candidate reservoir species ranges to assess their potential importance for MPXV infections in humans, and in projecting shifts in the geographic distribution of human MPX under climate change. Our models for MPXV occurrence under current climate conditions, and the importance of temperature and precipitation variables are broadly consistent with those found in previous studies [23], [45], providing confidence that our models under current climate conditions are useful for subsequent projection onto future climate variables, offering new insight in potential range shifts of human MPX.

Models for the occurrence of human MPX using reservoir species were nearly identical to those using only climate variables. This suggests that the MPXV and reservoir species share a unique habitat that is largely captured by environmental variables. Nevertheless, reservoir species are likely to be more biologically relevant with respect to the transmission of MPXV to humans. This is supported by the facts that MPX outbreaks have recently been recorded in Sudan [10], [46], where it might be endemic, and the US, where MPXV was incidentally introduced through imported African rope squirrels (*Funisciurus* sp.), dormice (*Graphiurus* sp.), and giant pouched rats (*Cricetomys* sp.), and subsequently transmitted to prairie dogs (*Cynomys* sp.) [47], [48], [49]. In these areas, where MPX has not been previously recorded, environmental conditions are highly dissimilar to those seen in the African tropical forest area, yet locally abundant and immunologically naive rodent populations may be effective MPXV reservoirs once exposed to the virus. This underlines the relation between human MPX and current and potentially new reservoir species as a function of their habitat requirements and shifts due to climate change. Despite the importance of reservoir species in MPXV transmission, it is plausible that certain environmental conditions directly favor the survival or proliferation of the virus itself. For instance, specific temperature and light regimes increase the time that pox virus survives outside a host (reviewed in [50]). Experimental studies suggested that transmission of MPXV was via bodily excrements [18], [19], [49] and it is likely that environmental conditions affect the efficiency of these transmission mechanisms.

### Important reservoir species

We used a set of candidate MPXV reservoir species to identify those that are potentially the most important. It is possible that species not included in our list of candidate reservoir species are also, or even more important reservoirs for MPXV, but our list contains most of the species that have previously been implicated in MPX occurrence (although not all of them have been shown to be infected with MPXV, and only one species (*F. anerythrus*) has been found infected in the wild [51]). In a human MPX model where reservoir species were based on both climate and remote sensing variables, presence of rope squirrels (*Funisciurus* sp.) was highly important in determining MPXV occurrence (Fig. 4). This is consistent with previous modeling [30] and serological screening results [19], [20]. When reservoir species distributions were based on climate variables only, a model for human MPX suggested that the pangolin *M. tetradactyla* and the monkey *C. wolfi* were important predictor variables, as well as the rope squirrel *F. pyrropus*, which contained unique information not present in other reservoir species distributions (Fig. 4). Yet, it remains unclear whether the pangolin *M. tetradactyla* is an important reservoir species, as there is little evidence for MPXV infections [8], and contact with pangolins was not identified as a risk factor for human MPX infection [9]. We speculate that its importance could be the result of shared habitat requirements with true reservoir species. Such cross-correlations between species' distributions could result in the false identification of potential reservoirs that are in fact not a reservoir for MPXV. They are, however, unlikely to result in false negatives, because species distributions that do not correlate with the distribution of human MPX cases will all be regarded of low importance. As such, our results provide a basis for identification of suspected reservoir species that should be targeted for intensive screening efforts, including the pangolin *M. tetradactyla*.

### Future projections of human MPX

Two of the key assumptions in the procedure of projecting current reservoir species distributions on future climate layers, and their subsequent use in modeling human MPX occurrence, is that current reservoir species will continue to be key reservoirs in the future, and that their environmental requirements will remain the same over the course of the next 50–100 years, which is the approximate time span used in many future climate models. These assumptions seem justified given that this period is relatively short to allow for major evolutionary responses to climate change in vertebrates. Nonetheless, the potential for MPXV to jump species boundaries into new hosts remains [52], subsequently affecting areas where our models project low MPXV suitability in the future. An additional assumption of our projections for human MPX is that the interaction between humans and reservoir species remains the same. If human populations in the future become less reliant on bush meat for protein, the number of human MPX cases may decrease – though this will be challenged by the continuing decline in population immunity as time passes since the cessation of smallpox vaccination. Conversely, if the dependence on bush meat remains at current levels or increases, it is plausible that the rise in human MPX cases [9] will continue in the future.

The consensus emerging from the eight global climate models considered here is that the geographic distribution of human MPX occurrence will change considerably across Tropical Africa. Areas in western Africa, where a less virulent strain of MPXV is present, are projected to become less suitable for the virus. Projections in West Africa are tentative, however, because only few recorded presence sites were available, and the West African MPXV strain may be associated with a different set of reservoir species, each with its own specific habitat requirements. Central regions in DRC and the Republic of Congo are also projected to experience lower levels of human MPX occurrence under future climate conditions. However, areas in the northern part of the contemporary range of human MPX will likely become more suitable as a result of changes in temperature seasonality (Bio4; mean annual standard deviation in temperature), which was an important variable in several reservoir species' niche models. In addition, projections derived from all included climate models suggest a shift eastward into areas where MPXV has not yet been recorded. Interestingly, and suggestive of the significance of our results, the incidence of human MPX in DRC has been shown to have increased over the past three decades beyond the level expected by the higher percentage of the population susceptible to smallpox, after cessation of the smallpox vaccination program in 1980 (unpublished). Although the cause of this increase remains unclear, it is concordant with the projected rise in suitability under changing climate conditions. Moreover, new reports confirm human cases of MPXV in southern Central African Republic associated with bushmeat consumption [53].

The question whether MPXV will be transmitted to humans in areas that will become more favorable to MPXV reservoir species is at least partially dependent on human behavior. For example, although our models suggest an increased risk for human MPX in Uganda in the future, Ugandan communities are generally more pastoral and less reliant on hunting animals that may act as MPXV reservoirs than communities in DRC. Hence, the spread of MPXV reservoirs into new areas may not necessarily result in higher incidence of MPX in humans, as long as contact between humans and reservoirs can be avoided. On the other hand, the projected increased suitability for MPXV in eastern DRC may have severe consequences for its human population. This is an area with high human population densities that, in contrast to communities in bordering Uganda, are largely reliant on bush meat for protein. Furthermore, the area's population is predicted to continue growing based on extrapolations from recent censuses using annual growth rate estimates (Fig. 3E; [54]). In particular, the projected population structure indicates a high proportion of individuals under 20 years old, which are at increased risk of MPX. For example, boys have the highest incidence of MPX, probably due to hunting behavior [55]. Although projections for human population growth at spatial scales smaller than the country size are highly uncertain for periods beyond 2015, it seems reasonable to suspect that growth will continue in areas where high growth rates are currently projected. According to United Nations projections, by 2050 there will be a 36% increase in male children in DRC compared to 2009 numbers [56]. In light of this, an epicenter of future MPX outbreaks and increased human-to-human transmission could potentially arise in eastern DRC. This situation is aggravated by the civil unrest in the region. First, the unstable situation may cause people to move from urban to rural areas and into the forest, where contact with animals infected with MPXV is more likely. Second, health care may be poor or non-existent. Third, surveillance in areas of civil unrest is severely limited, as was the case during the 1996 to 1997 outbreak of what was potentially human MPX [17] or chickenpox [57]. Although our future projections are for a period 40–80 years from now, there is no clear indication that the trend of increasing human MPX incidence during the past 30 years will cease in the near future. Thus, there is a need for continued and increased monitoring of human MPXV infections, as well as more detailed efforts to identify reservoir species and characterize their ecology, and to understand the risk factors underlying animal-to-human transmission. Strategies to reduce the risks of MPX transmission to humans should be developed and implemented. Unfortunately, the places where MPX occurs most frequently are in villages that are remote and difficult to access. Disease surveillance is challenging even for relatively well funded programs, and the development of a functioning health system is of utmost importance, yet complex and not easily resolved. Before now, little was known about potential range shifts of MPXV under climate change. By identifying high-risk areas, we hope that the limited resources can be utilized more effectively. At the same time, it is essential to understand the risk posed by human-to-human transmission as the population grows and becomes more susceptible.

Our finding that deforestation has been a significant driving factor of MPX transmission in Sankuru suggests that, in addition to climate change, land use changes may also influence reservoir species distributions and abundance. The majority of the areas where human MPX is projected to increase are covered in forest, where the putative reservoir species naturally occur (Figs. S8, S9). Land conversion in these areas is rampant, where primary forest is turned into secondary forest due to timber extraction, or into agricultural fields. This type of land cover change may result in increased habitat suitability for some key reservoir species. For instance, rope squirrels (*Funisciurus* sp.), potentially one of the most important primary hosts, have been reported to be more abundant in converted areas that are heavily used by humans than in primary forest [18]. These may also be areas where other rodents are more abundant than in primary forests, due to higher availability of their major food sources, seeds and seedlings. This may have consequences for the distribution of MPXV in the future, as continued deforestation may result in range expansions or higher abundance of important reservoir species. The complex relationship between virus, reservoirs, and environmental conditions further emphasize the need for detailed studies regarding viral and host ecology.

In sum, our study contributes to a better understanding of the potential impacts of climate change and deforestation on the distribution of human MPX in Tropical Africa. Our results support previous findings that rope squirrels (*Funisciurus* sp.) may be important MPXV reservoirs. In addition, our models also suggest that the monkeys *C. wolfi* and *C. albigena*, as well as locally abundant pangolin (*M. tetradactyla*) merit investigation as potential reservoirs. Although we have not established these species as MPXV reservoirs based on active screening, our models suggest that closer investigation of these species is warranted. In contrast, Gambian rat (*C. gambianus*), greater cane rat (*T. swinderianus*), and tree pangolin (*M. tricuspis*) are not supported as likely reservoirs by our model. Our predictive maps of potential future MPX occurrence are helpful in identifying areas with particularly suitable future environmental conditions for human MPX reservoirs, which may be high-priority areas for monitoring by public health decision-makers. Areas where the probability of MPXV occurrence is projected to increase should be closely monitored. In addition, the identification of locally abundant potential reservoir species and behavioral risk factors will be helpful in assessing the human health hazards imposed by shifts in the range and prevalence of MPXV as a result of changing environmental conditions.

## Materials and Methods

### Assessing the effects of present-day climate and forest clearing on MPX outbreaks at the local scale in DRC

**Figure 6:**
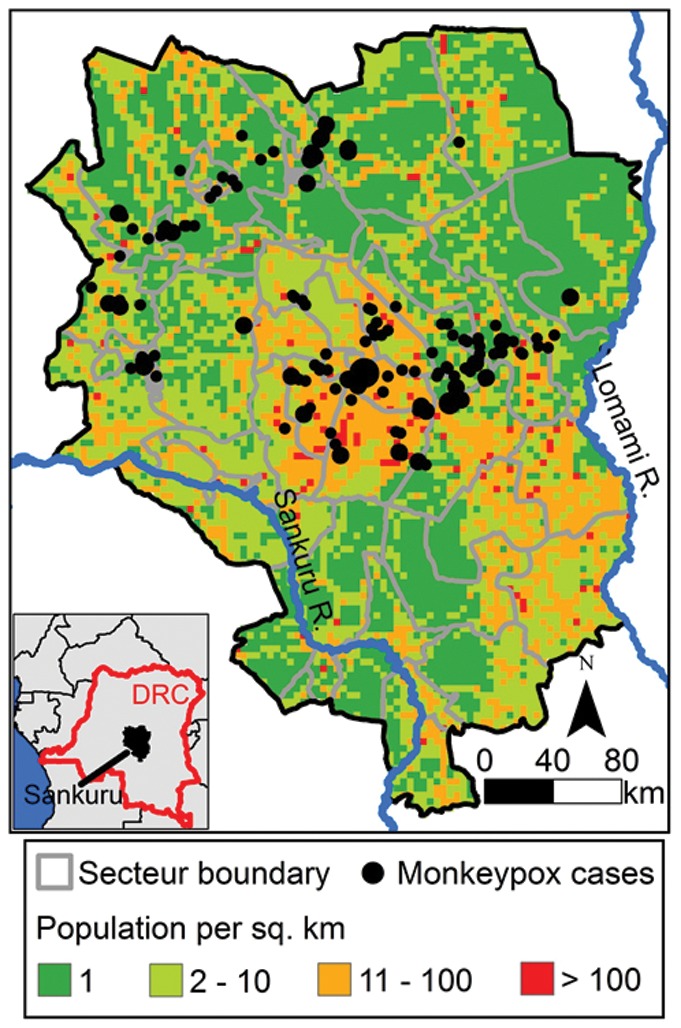
Sankuru, DRC. Site of the local scale study of the effects of climate and deforestation on MPX occurrence.

To investigate the contributions of recent climate change and deforestation to the transmission of MPX, we carried out a local scale study in Sankuru, DRC (Fig. 6). We selected Sankuru because MPX surveillance has been carried out recently in the district [9] and maps of forest clearing since 2000 are also available for the area [58]. Thus, deforestation is defined as a relatively recent event. In DRC, and over such a recent time frame, deforestation typically does not result in urbanization, but in either secondary-growth forest or agricultural landscapes. It is these kinds of landscape where close contact between humans and reservoir species is likely to be most frequent. Next, we analyzed remotely sensed data to generate maps of Sankuru's climate over the past ten years (Fig. S6), developing maps of temperature and canopy moisture (Table 6). We examined these climatic variables because temperature and wetness are important ecological drivers of rodent-borne diseases in DRC [23], [59]. We also calculated the correlation between drought and the Atlantic Multidecadal Oscillation (AMO; see Text S1). This provides an indirect measure of the strength of recent climate change in Sankuru [41]. We combined the climatic data with maps of deforestation in Sankuru to estimate the relative importance of forest clearing and climate as drivers of MPX infections in humans.

**Table 6:** Climatic and land cover variables used to analyze MPX transmission.

Scale	Variable	Year(s)	Satellite	Reference(s)
Sankuru	Canopy moisture	2000–09	QuikSCAT	[71]
	Correlation between MWD and AMO	1998–2010	TRMM	[86]
	Deforestation	2000–10	Landsat	[58]
	Temperature of the coldest quarter	2000–10	MODIS	[87], [88]
	Temperature of the warmest quarter	2000–10	MODIS	[87], [88]
Tropical Africa	Vegetation greenness (NDVI)	2001–05	MODIS	[89]
	Percent tree cover	2001–05	MODIS	[70]
	Canopy moisture	2000–09	QuikSCAT	[71]
	Elevation	2000	SRTM	[90]
	Mean temperature (Bio 1)	1950–2000	Interpolated from weather stations	[67]
	Temperature range (Bio 2)	1950–2000	Interpolated from weather stations	[67]
	Temperature seasonality (Bio 4)	1950–2000	Interpolated from weather stations	[67]
	Temperature of warmest month (Bio 5)	1950–2000	Interpolated from weather stations	[67]
	Mean precipitation (Bio 12)	1950–2000	Interpolated from weather stations	[67]
	Precipitation seasonality (Bio 16)	1950–2000	Interpolated from weather stations	[67]
	Precipitation of the driest quarter (Bio 17)	1950–2000	Interpolated from weather stations	[67]

AMO = Atlantic Multidecadal Oscillation, MWD = maximum water deficit, NDVI = normalized difference vegetation index.

Our objective was to identify regions with significantly more MPX cases in humans than would be expected by chance (hereafter “MPX hotspots”). Analyses were carried out at the level of administrative regions within Sankuru called “secteurs” (Fig. 6). The climate and deforestation data were for this purpose aggregated to the scale of secteurs in ArcGIS 9.3 (ESRI, Redlands, CA) and utilized as covariates in a statistical model implemented in the SaTScan 9.0 software package SaTScan tests the null hypothesis that the number of MPX cases in each secteur is Poisson distributed and proportional to human population size [31], [32], [60], [61]. The alternative hypothesis is that the risk of MPX within a secteur or secteurs is elevated compared to other secteurs. SaTScan searches for secteurs that have anomalously high risk of MPX by scanning an elliptical window across Sankuru and calculating the number of observed and expected cases inside the window. A variety of ellipses with various angles and shapes were tested. The MPX primary hotspot is the window with the maximum likelihood and the secondary hotspot is the window with the second highest likelihood (see Kulldorff [60] for the likelihood function). A hotspot is an area that has a significantly higher number of disease cases than would be expected by chance, while accounting for the geographic area of the hotspot and covariates [60], [62], [63]. In our analyses, each hotspot typically contained several secteurs, but there was only one primary hotspot and one secondary hotspot. We compared the models that included climate, deforestation, and their interaction to a reference model with no covariates.

### Assessing the role of present-day climate on MPX outbreaks at the regional scale in Tropical Africa

Next, we expanded the local scale study to map the geographic distribution of MPX throughout Tropical Africa. Like the local scale study in Sankuru, our analysis at the regional scale utilized climatic variables. However, this analysis did not consider deforestation because maps of forest clearing are not available for the entire region. In addition, the regional scale study examined the geographic distributions of wildlife hosts to predict MPX risk.

We used ecological niche modeling techniques to model reservoir species and human MPX distributions across Tropical Africa, with special emphasis on Central Africa. We created a total of 198 models with different combinations of predictor variables for candidate reservoir species and human MPX in a two-step approach (Fig. 1): 1) model reservoir species distributions using environmental data (Table 6) under current and future climate conditions, and 2) use the predicted current and projected future reservoir species distributions to model the distribution of human MPX. Because future projections are entirely based upon predicted changes in the climate, and do not take into account land cover changes, we first ran models for contemporary climate conditions excluding remotely sensed habitat characteristics to establish the current climatic determinants of reservoir species occurrence. These climate-only models were compared to models including both climate as well as remote sensing variables to assess the influence of omitting remotely sensed habitat variables, and the validity of the climate-only models and future projections. Because remote sensing variables may add valuable information to the distribution models, future projections (see below) were considered to be useful only when models using current climate broadly recovered the same distribution of reservoir species as models using both current climate as well as habitat. We expected to see that climate-only models would show a more generalized and potentially slightly broader distribution due to the relatively low native resolution and less small-scale spatial heterogeneity of the climate variables compared to remotely sensed data layers.

After modeling the distributions of candidate reservoir species, we modeled human MPX distributions under current conditions and assessed the concordance among models using the following predictor datasets (Fig. 1): 1) reservoir species distributions (based on climate and remote sensing variables) and in addition the climate and remote sensing variables (the full model), which may contain additive information on top of the reservoir species distributions that were also based on both types of data; 2) only reservoir species distributions (based on climate and remote sensing variables); 3) reservoir species distributions (based on climate variables) plus climate variables; and 4) only reservoir species distributions (based on climate variables). To model future human MPX distributions under different climate change scenarios, we projected reservoir species distributions onto future climate variables. We then used the results as input for models of human MPX under approaches 3) and 4) above, since future remote sensing variables are not available. Below, we describe the data acquisition and modeling approaches in more detail.

### Human MPX occurrence data

Human MPX occurrence data for this study was partly collected during an ongoing study in DRC and partly derived from a World Health Organization (WHO) based intensified disease surveillance program during the 1970s and 1980s. Since 2001, UCLA, in collaboration with the WHO, the National Institute of Biomedical Research, and the Ministry of Health in Kinshasa have been actively conducting MPX disease surveillance in humans in DRC. As part of this screening effort, disease surveillance has been intensified since 2004. The study methods are described in detail in [9], [64]. Briefly, we initiated an intensified active disease surveillance program in 12 health zones of the Sankuru district. This district was selected for intensified surveillance because the majority of reported MPX cases since 2001 were in this area, and it was a region of epidemiologic priority for the WHO from 1981–1986. The northern part of the district consists mainly of lowland tropical forest, while the southeast has a more varied terrain characterized by ecotone habitats consisting of mosaics of forest, grassland, and woody savannah. The economy of Sankuru is largely based on traditional agriculture and hunting. As a consequence, contact of people with the natural environment is intimate: the majority of villages, surrounded by traditional fields, are located in clearings in the forest, and virtually all protein is obtained from hunting locally available wild animals, of which monkeys and rodents are among the most commonly hunted species.

The surveillance effort was relatively even across the Sankuru district. Trained field teams traveled regularly in their respective health zones, encouraging local communities to report suspected cases of MPX and examining and documenting reported cases. Crusted scabs and vesicle fluids were inoculated onto MA104-cells, and presence of MPXV in samples was confirmed by sequencing the entire open reading frame of the hemagglutinin (HA) gene. The home village of each suspected MPX case was noted and its location later georeferenced by GPS. Ethical approval was obtained from the Committee on Human Research at UCLA School of Public Health and the Institutional Review Board at the Kinshasa School of Public Health (KSPH). Informed consent (and child assent in children 5–18 years of age) was obtained verbally from all participants and legal guardians of children in French and Otetela (the common local language spoken in the area). Consent was obtained verbally given the low rate of literacy in the region. Consent was documented by participants either by signature or their mark and confirmed by a witness. The verbal consent process was approved by both institutional review boards at UCLA and KSPH.

In addition to these localized data, we used locality information from human MPX cases reported by the Centers for Disease Control (CDC) and WHO in the 1970s and 1980s as part of the smallpox eradication program, and previously used in [23]. Combining data sets from the two time periods is justified in terms of our predictors, as the climate variables used are based on 50-year averages, spanning 1950–2000. Cases from the data set from the 1970s and 1980s that could not be georeferenced accurately due to incomplete or confounding location information were omitted. Location assignments for the remaining cases were accurate to at least 1 minute (∼2 km) [23]. Because the combined datasets resulted in high clustering of human MPX cases in the Sankuru District, potentially biasing the distribution models to areas with high sample density, we resampled the data, and only retained sites that were at least 10 km apart, resulting in a total of 93 presence-sites. The 10 km threshold was used, because it is an order of magnitude larger than the resolution of our environmental variables, and resulted in greatly reduced clustering of human MPX positive sites. Selection of sites at distances under 10 km to be omitted from the final dataset was done at random. Because the climate variables used in the spatial predictions of reservoir species tend to vary at distances >10 km, it is unlikely that our subsampling procedure would result in a reduction of the environmental niche space included in the final dataset. Moreover, inaccuracies in georeferencing in the order of a few kilometers are unlikely to have an effect on our results.

### Reservoir species data

Potential reservoir species (Table 2) were identified from previous studies showing MPXV infections in these species, or studies suggesting these species might be important in transmitting MPXV to humans [17], [18], [19], [20]. Point locality occurrence data of potential reservoir species were collected as part of our MPX screening efforts, as well as from museum data, compiled in the online Global Biodiversity Information Facility (GBIF; http://www.gbif.org/ Accessed April 2010) and Mammal Networked Information System (MaNIS; http://manisnet.org/ Accessed April 2010) databases. Suspected MPXV-positive human subjects (see above) were asked to fill out a questionnaire, which included questions regarding recent and regular exposure to potential MPX reservoir species. For each potential reservoir species, localities from the questionnaires were added to museum data. To avoid a bias towards the Sankuru District due to high sample density in that area, we restricted the number of localities from the questionnaires to 10–15 per reservoir species. Since species identification by human subjects could not be validated, we also ran models without localities from the questionnaires. These models did not differ from those where localities from questionnaires were included, and are therefore not shown.

Museum records that were collected before 1940, or that were geo-referenced to only 0.1 decimal degree, were omitted from the final datasets used to predict the reservoir species' distributions. The use of museum records could result in inaccurate ecological niche models if they are imprecisely georeferenced or when land conversion has occurred at the sampling locality since the time of collection [65], [66]. However, because our primary focus was on climate variables comprising 50-year averages (see below), and showing broad rather than small-scale spatial heterogeneity, we deemed the use of museum records appropriate. As an additional check of the accuracy of the museum records, a comparison was made to species distribution maps from the International Union for Conservation of Nature (IUCN; www.iucnredlist.org Accessed May 2010), estimated using expert knowledge. Records that were outside the ranges shown on these maps were considered to be of low confidence and were omitted from the final input data set. This procedure also at least partially mitigates issues resulting from new taxonomical insights and splitting of a single species into two or more separate species to the extent that the new taxonomy has been implemented in the IUCN database. Nevertheless, records may still include those of two closely related species. In such a case, models would likely present a wider niche than the one suitable for just one of the two sister species. However, species that are closely related may likely both be potential reservoirs of MPXV, and the possibility that models show slightly wider niches was not regarded a major concern for their subsequent use in modeling human MPX occurrence.

Few readily available museum records existed for the following potential reservoir species: the long-tailed pangolin (*Manis tricuspis*), tree pangolin (*M. tetradactyla*), Demidoff's galago (*Galagoides demidoff*), Wolf's monkey (*Cercopithecus wolfi*), and the grey-cheeked mangabey (*Lophocebus albigena*). Although it was suggested that these species might carry MPXV, they have not been implicated as species that might act as key reservoirs in which the virus continues to exist. We resampled downloaded ecological niche models for these species from the African Mammals Databank (AMD; http://www.gisbau.uniroma1.it/amd/ Accessed April 2010) and built new distribution models using the same set of environmental variables used for the species with good coverage of museum records (see below). The AMD species distributions are the result of a combination of expert knowledge on extent of occurrence and ecological requirements, modeled species-environment relationships, and model validation at 400 sites where species presence or absence was recorded in the field. To resample the downloaded distribution models, in ArcGIS 9.3 (ESRI, Redlands, CA) we created 2500 random points across Tropical Africa, and extracted the probability values of species occurrence. For each species, we subsequently used the 100 localities with the highest probabilities to model its distribution, and visually compared the level of concordance between our results and the original AMD maps. For each species, the localities that we used fell within the top 3% of suitability.

### Contemporary climate and remote sensing variables

We compiled a set of moderately high-resolution climate and satellite remote sensing variables to characterize the various habitat types in Tropical Africa (Table 6). These included eight out of a total of 19 bioclimatic variables (for the procedure to reduce the number of variables, see below) from the WorldClim database [67], which are spatially explicit estimates of annual means, seasonal extremes and degrees of seasonality in temperature and precipitation based on a 50-year climatology (1950–2000), and have been shown to represent biologically meaningful variables for characterizing habitat requirements [68].

In addition to these ground-based measurements of climate, we used satellite remote sensing data from both passive optical sensors (MODIS; https://lpdaac.usgs.gov/lpdaac/products/modis_overview/ Accessed June 2010) and active radar scatterometers (QuikScat; http://www.scp.byu.edu/data/Quikscat/SIRv2/qush/World_regions.htm Accessed June 2010) to infer a broad spectrum of ecological characteristics of the land surface. From the MODIS archive, we used the monthly Normalized Difference Vegetation Index (NDVI) to infer vegetation density [69]. In addition, we used the vegetation continuous field [70] product as a measure of the percentage of tree cover. From QuikScat (QSCAT), we obtained monthly raw backscatter measurements that capture attributes related to surface moisture and roughness [71], and from the Shuttle Radar Topography Mission (SRTM), we used elevation data. Time series of the remote sensing data sources were acquired to roughly match the period of field sampling (QSCAT and tree cover from 2001; NDVI data represent means over 2000–2004). Variables with native resolutions higher (e.g. SRTM: 30 m) or lower (e.g. QSCAT: 2.25 km) than 1 km were reaggregated to a 1 km grid cell resolution in ArcGIS 9.3 (ESRI, Redlands, CA) Spatial Analyst with the ‘resample’ and ‘aggregate’ functions respectively (Table 6). This resolution is often used in ecological niche modeling at the regional or sub-continental scales, and balances resolutions from climate data, which often have coarser native resolutions, and remote sensing data, with higher native resolutions.

To improve interpretation and avoid overfitting due to cross-correlations among environmental variables, we checked for covariance among variables, and only included those with substantial unique variance (with Pearson's correlations <0.9). Various criteria were used to decide which layers of correlated pairs were retained for further analysis. These included keeping layers that are more commonly used in distribution modeling (WorldClim) or that exhibit larger contrast/variance over the study area (QSCAT) as well as having best data quality (NDVI). To assess whether the spatial resolution (i.e. the level of aggregation) influenced the cross-correlation among environmental variables, we also checked for correlations between variables at 5 km and 25 km resolutions. The results were highly similar to those obtained from correlation analyses at 1 km resolution, and none of the variables selected based on 1 km resolution analyses were correlated with Pearson's correlations >0.9.

### Projections under future climate change

To assess the potential impact of future climate change on the distribution of MPXV in Central Africa, we first projected the current relationship between reservoir species and climatic conditions onto predicted future climate layers. In a second step, we projected the current relationship between MPX occurrence and reservoir species onto the projected future reservoir species distributions. To determine the range of potential shifts in MPXV distributions under different climate change scenarios, sixteen climate models from the IPCC 4^th^ Assessment Report with differing climate sensitivities and IPCC–SRES greenhouse gas emission scenarios were used for 2050–2060 and 2080–2090 (Table S1). These IPCC projections were statistically downscaled to 1 km resolution [67] and made available by the International Centre for Tropical Agriculture (http://ccafs-climate.org/ Accessed September 2010) [44]. As the confidence intervals around climate change scenarios tend to become broader with predictions further into the future, we included the 2050–2060 predictions. Nevertheless, as the 2080–2090 predictions represent more extreme climate change scenarios, these were also included in our study. In fact, the 2080 predictions of atmospheric CO_2_ concentrations may be reached much sooner, as current emissions already exceed the trajectories of the highest scenarios [72], [73], [74]. Thus, projections of human MPX occurrence on the 2080–2090 climate scenarios may be relevant for purposes of our study.

A comparison among the IPCC 4^th^ Assessment climate projections shows that the direction and magnitude of the future projections are relatively consistent in our study area. Between 14–18 out of the 21 models (IPCC 4^th^ Assessment Report) agreed on an increase in annual precipitation in the Congo Basin, lending credibility to these projections. The multi-model ensemble (based on 21 models) from the IPCC 4^th^ Assessment Report predicts approximately 3°C temperature increase and 0–10% increase in annual precipitation in our study region.

### Distribution modeling

To model the spatial heterogeneity in reservoir species and MPXV occurrence, we used the Maximum Entropy approach, a machine learning technique implemented in Maxent 3.3.3a [75]. It relies on the assumption that the incomplete empirical probability distribution of occurrence (based on known occurrences) can be approximated with a probability distribution of maximum entropy (the Maxent distribution) subject to certain environmental constraints (based on all environmental variables in the model), and that from this distribution the potential geographic distribution of the group under study can be determined. Rather than relying on both recorded presence and absence data points, Maxent uses presence-only input data, and subsequently selects a set of random background points. This makes the algorithm well-suited for our purposes since we did not have absence data. The input data consist of a set of environmental layers for the study region and the observed disease presence localities within that region. Maxent then uses these data to estimate the environmental niche space that accurately describes the observed occurrences. Its predictions are continuous logistic probabilities with increasing values referring to more suitable habitats. In several multi-model comparisons, Maxent was shown to perform well with few point localities [76], [77], and in comparisons of ecological niche modeling techniques its performance was generally rated among the highest [77], [78]. We used the following settings of Maxent: 10000 background points; only linear or auto features; regularization multiplier = 3.0; maximum iterations = 500; convergence threshold = 0.00005. To assess model fit as a function of the different sets of input predictor variables, we used the Akaike Information Criterion corrected for sample size (AICc), implemented in ENMTools [79].

For modeling purposes, a location was considered to be positive for human MPX if one or more cases had been identified there. Localities of active human MPX cases used in modeling are shown in Fig. 3a. The number of sites used in each model for reservoir species and human MPX is shown in Tables 2 and 3. To examine whether predicted future environmental conditions in our study region are more extreme than those observed under current climate, we used the clamping option implemented in Maxent.

### Variable importance

To assess the relative importance of predictor variables in determining the distribution of human MPX, we used the variable jackknifing procedure implemented in Maxent. Using a subset of the original variable set, new models are computed and their performance as measured by the regularized training gain (the average log probability of the presence samples, corrected for a uniform distribution with gain = 0) compared to that of the full model [75]. Two ways of selecting a subset of variables are implemented. First, each variable is used on its own, providing an assessment of the information content of the variable itself. Second, models are computed using all but one predictor variable, which is useful in assessing the amount of unique information in the omitted variable. This jackknifing approach is particularly helpful when some predictor variables (e.g., reservoir species distributions) are cross-correlated, because they will be nearly equally important in determining the range of human MPX cases.

### Model performance

The area under the receiver operator curve (AUC), implemented in Maxent, was used as a first-order assessment of model performance, where an AUC score of 0.5 indicates random prediction, and a score of 1 a perfect prediction. In addition, the available data were split into training and test datasets, where the former is used to estimate parameters for (“train”) the model using the settings from the full model (with the lowest AICc in the case of human MPX), and the latter to test the predictions of that model. Here, we used two different approaches to generate different training and test datasets. First, we ran additional models for each predictor data set with a random test percentage of 40% and 500 iterations for each model, as implemented in Maxent. We considered model performance high if test AUC values were >0.75 [80]. This procedure randomly selects the training data from the original dataset, and training and test sites may thus be spatially autocorrelated. To address this issue of spatial autocorrelation, in a second approach, we divided the study region in two spatial partitions, one of which was used as training data and the other as test data (Text S2). In this case, training and test datasets are unlikely to be spatially autocorrelated, in contrast to the situation when a random subset is taken to serve as test data. While there may be many ways to create a spatial subdivision of the dataset, we focused on one that generated nearly even sampling sizes in training and test datasets in a west-east split. We modeled reservoir species and human MPX occurrence across the entire region, using only the training data (east or west), and compared the projections to the corresponding observed test data.

The use of AUC values unaccompanied by other means of assessing model performance in species distribution modeling has been shown to be problematic [81], [82]. Therefore, to further ensure that our model validation was robust, we used a threshold-dependent approach for the comparison of predicted versus observed occurrences for each model, in which the logistic probability output was converted to a presence-absence map based upon an empirical threshold [83]. We used the ‘balance threshold’ from Maxent, which balances the training omission rate and the proportional predicted area, two measures of quality of a binary prediction [75]. With this threshold, we calculated the proportion of the total area of our study region where human MPX was predicted to be present, and tested whether more test localities were predicted to be positive for human MPX than expected based upon the proportional predicted area using a one-tailed binomial test [84]. Thus, the expectation for the null (a random prediction) is that the proportion of sites predicted to be positive for MPXV is equal to the proportion of the total area where MPXV is predicted to be present. For predictions of human MPX, we also used a threshold-independent approach, where we extracted the logistic probability scores for localities that were used as training sites in one model and test sites in a second model, in which the data partitioning was reversed. That is, the logistic probability values of western sites based on a model using western sites for training were compared to those based on a model with eastern sites for training. Similarly, the probability values of eastern sites were compared between models using eastern versus western sites for training. Models were then compared using a Wilcoxon signed-rank test, expecting to see no significant differences if the training sites accurately predicted the test sites. All statistical tests were carried out in the R statistical framework [85].

As a final means to evaluate whether the modeled species distributions were meaningful, we visually compared modeled species ranges with range maps based on expert knowledge, available from the IUCN Red List (IUCN; www.iucnredlist.org Accessed May 2010), Mammal Networked Information System (MaNIS; http://manisnet.org/ Accessed April 2010), African Mammals Databank (AMD; http://www.gisbau.uniroma1.it/amd/ Accessed April 2010), and from [33] and [34]. Expert-based maps often show only the range limits of species, whereas species distribution models offer the advantage that they also show the gaps within those outer boundaries. In all cases, modeled distributions showed range limits very similar to those based on expert knowledge.

## Supporting Information

Figure S1 **Variable importance of Maxent predictions for reservoir species distributions.** Results are shown for tests in which only the variable in question was entered into the model (dark blue bars) and in which all variables except the one in question were entered (light blue bars). Longer dark blue bars and shorter light blue bars indicate higher variable importance. Bio 1: mean annual temperature; Bio 2: daily temperature range; Bio 4: temperature seasonality; Bio 5: maximum temperature of the warmest month; Bio 12: mean annual precipitation; Bio 16: precipitation seasonality; Bio 17: precipitation of the driest quarter.
(TIF)

Figure S2 **Predictive models for MPXV reservoir species in Tropical Africa.** Maxent predictions of MPXV reservoir species occurrence under contemporary climate conditions, using climate variables as predictor, and averages for eight climate change scenarios each for the periods 2050–2060 and 2080–2090.
(PDF)

Figure S3 Predictive models for human MPX in Tropical Africa. Maxent predictions of human MPX occurrence under contemporary climate conditions, using different environmental variable sets as predictors: reservoir species (based on climate and remote sensing variables) plus climate and remote sensing variables; reservoir species (based on climate and remote sensing variables); reservoir species (based on climate variables) and climate variables; reservoir species (based on climate variables). For each model, all ‘features’ were allowed to be used in Maxent (“auto features”, i.e. linear and quadratic coefficients can be used for each predictor, as well as step functions and interactions).
(TIF)

Figure S4 **Future projections for human MPX occurrence under different climate change scenarios for 2050 (first eight panels) and 2080 (second set of eight panels).** Current reservoir species distributions were first projected onto future climate variables. The resulting future reservoir species distributions were subsequently used to estimate the future human MPX distributions.
(TIF)

Figure S5 **Spatial distribution of MPX occurrences in Sankuru showing overlap with (a) land cover types and (b) areas deforested since 2000.**
(TIF)

Figure S6 **Climatic and land cover variables used in the local scale study of MPX transmission.** (a) correlation between maximum water deficit (MWD) and the Atlantic Multidecadal Oscillation (AMO), (b) temperature of the wettest quarter, (c) temperature of the coldest quarter, (d) canopy moisture defined as maximum K_u_ band radar backscatter minus minimum backscatter (units: dB), (e) forest cover. The polygons outlined in black in panel (a) represent secteurs identified as primary or secondary hotspots of MPX.
(TIF)

Figure S7 **Association between climate and MPX genotypes in DRC at the national scale.** MWD = maximum water deficit. AMO = Atlantic Multidecadal Oscillation.
(TIF)

Figure S8 **Land cover map of our study area.** Hatched areas indicate a predicted increase in human MPX occurrence under the multi-model ensemble climate change scenarios for 2050–2060 (horizontal) and 2080–2090 (vertical).
(TIF)

Figure S9 **Plots of percent tree cover versus the predicted average increase in probability of human MPX occurrence for 2050 and 2080.** Small increases in human MPX occurrence are predicted in both savanna and forest habitat types, but the largest increases are mainly located in forest areas.
(TIF)

Table S1 Intergovernmental Panel on Climate Change (IPCC) scenarios examined for Central Africa.
(DOCX)

Text S1 **Estimation of Maximum Water Deficit, a measure of canopy moisture, in central DRC using remote sensing.**
(DOCX)

Text S2 **Spatial autocorrelation and spatial dependence.**
(DOCX)
