# Supplementary figures and images for: Pathogen-Host Associations and Predicted Range Shifts of Human Monkeypox in Response to Climate Change in Central Africa
Source: PLoS One. 2013 Jul 31;8(7):e66071. doi: 10.1371/journal.pone.0066071 (PMC3729955; doi:10.1371/journal.pone.0066071)

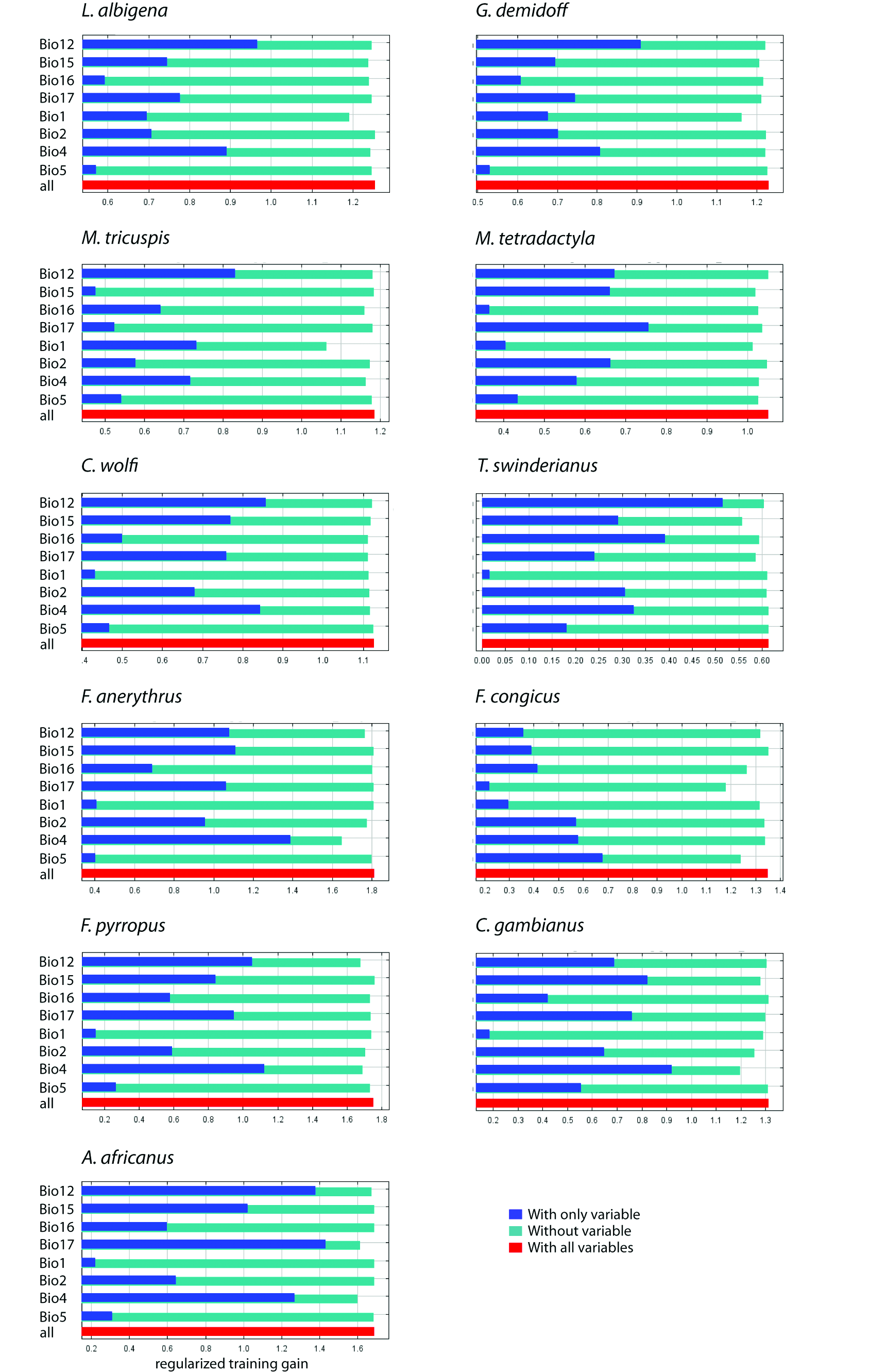

Supplement: Figure S1 — Variable importance of Maxent predictions for reservoir species distributions. Results are shown for tests in which only the variable in question was entered into the model (dark blue bars) and in which all variables except the one in question were entered (light blue bars). Longer dark blue bars and shorter light blue bars indicate higher variable importance. Bio 1: mean annual temperature; Bio 2: daily temperature range; Bio 4: temperature seasonality; Bio 5: maximum temperature of the warmest month; Bio 12: mean annual precipitation; Bio 16: precipitation seasonality; Bio 17: precipitation of the driest quarter. (TIF) [file pone.0066071.s001.tif]

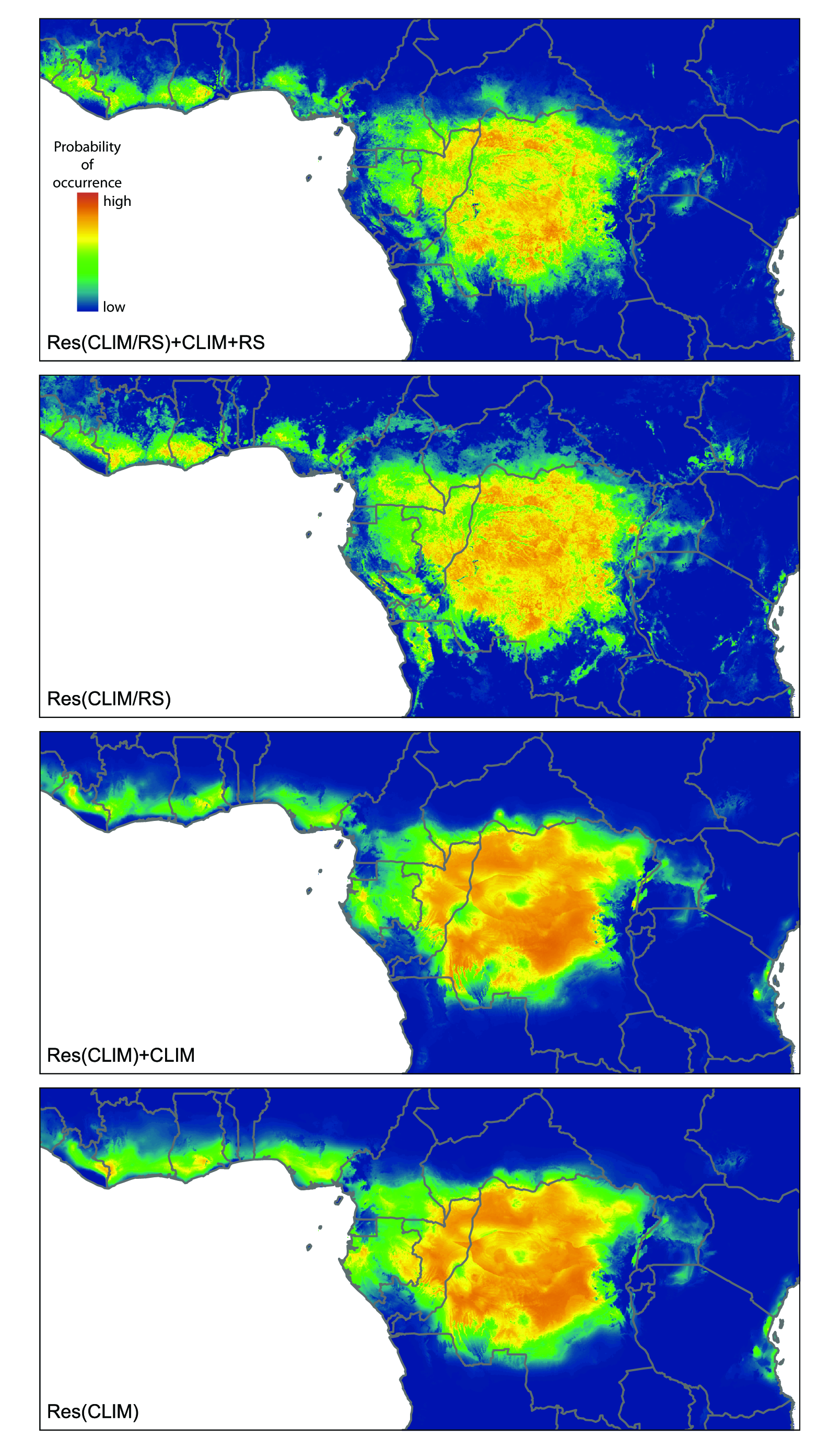

Supplement: Figure S3 — Predictive models for human MPX in Tropical Africa. Maxent predictions of human MPX occurrence under contemporary climate conditions, using different environmental variable sets as predictors: reservoir species (based on climate and remote sensing variables) plus climate and remote sensing variables; reservoir species (based on climate and remote sensing variables); reservoir species (based on climate variables) and climate variables; reservoir species (based on climate variables). For each model, all ‘features’ were allowed to be used in Maxent (“auto features”, i.e. linear and quadratic coefficients can be used for each predictor, as well as step functions and interactions). (TIF) [file pone.0066071.s003.tif]

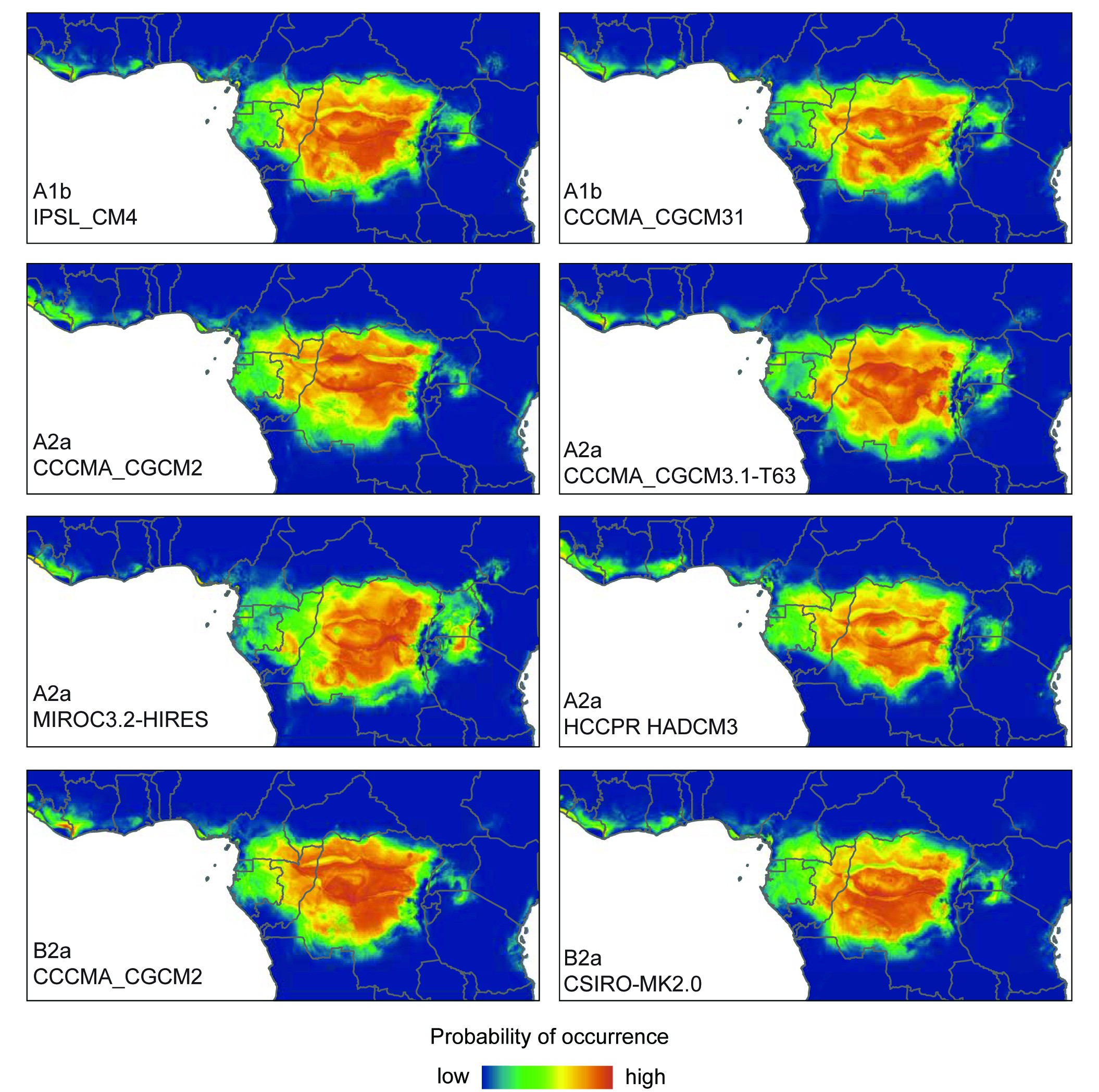

Supplement: Figure S4 — Future projections for human MPX occurrence under different climate change scenarios for 2050 (first eight panels) and 2080 (second set of eight panels). Current reservoir species distributions were first projected onto future climate variables. The resulting future reservoir species distributions were subsequently used to estimate the future human MPX distributions. (TIF) [file pone.0066071.s004.tif]

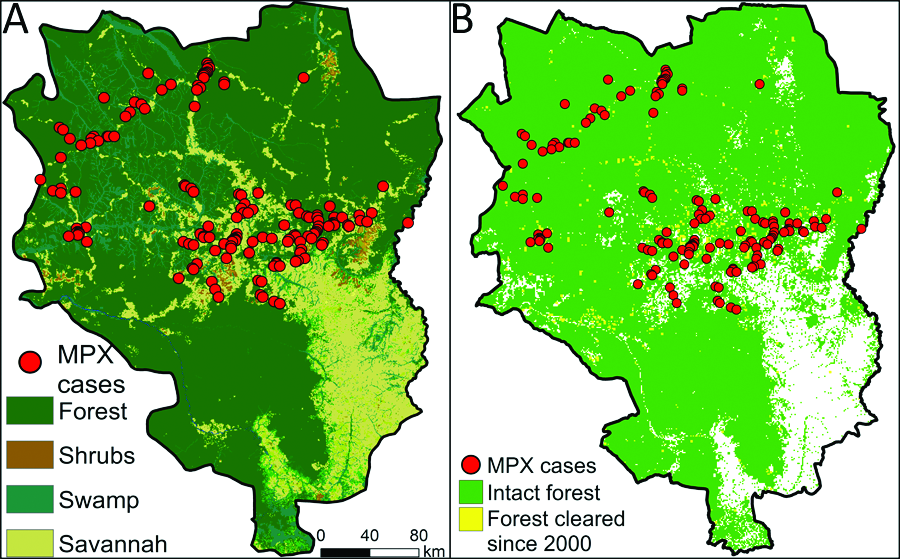

Supplement: Figure S5 — Spatial distribution of MPX occurrences in Sankuru showing overlap with (a) land cover types and (b) areas deforested since 2000. (TIF) [file pone.0066071.s005.tif]

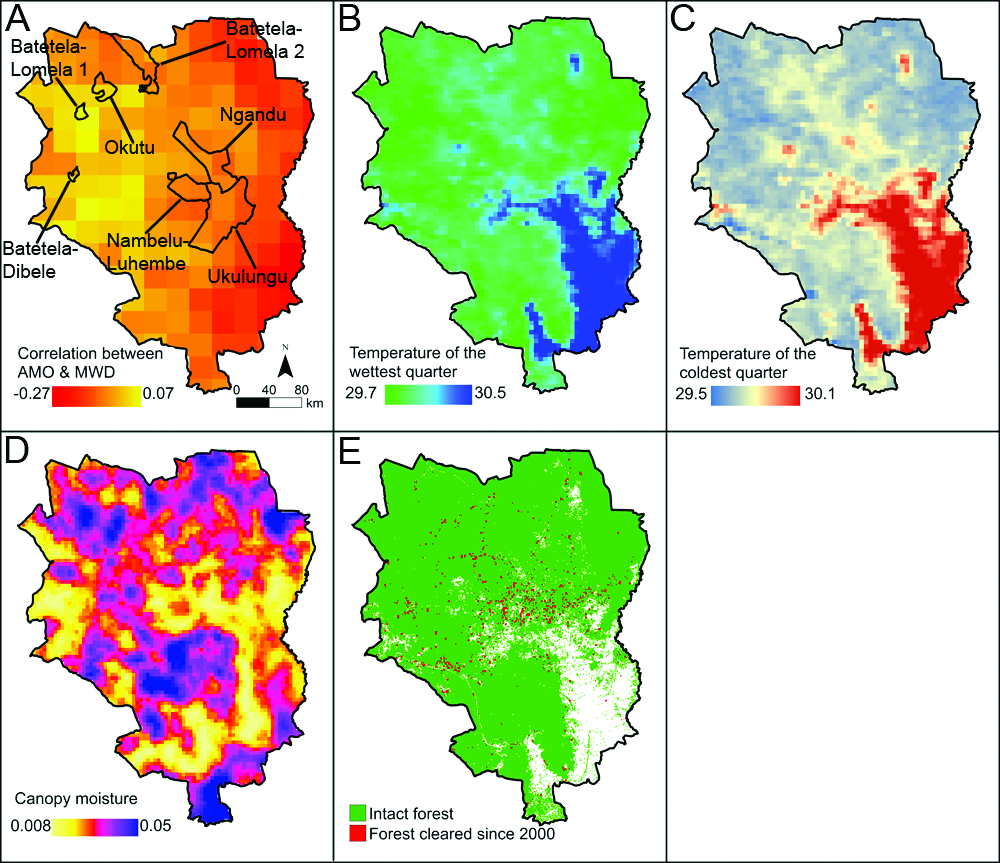

Supplement: Figure S6 — Climatic and land cover variables used in the local scale study of MPX transmission. (a) correlation between maximum water deficit (MWD) and the Atlantic Multidecadal Oscillation (AMO), (b) temperature of the wettest quarter, (c) temperature of the coldest quarter, (d) canopy moisture defined as maximum Ku band radar backscatter minus minimum backscatter (units: dB), (e) forest cover. The polygons outlined in black in panel (a) represent secteurs identified as primary or secondary hotspots of MPX. (TIF) [file pone.0066071.s006.tif]

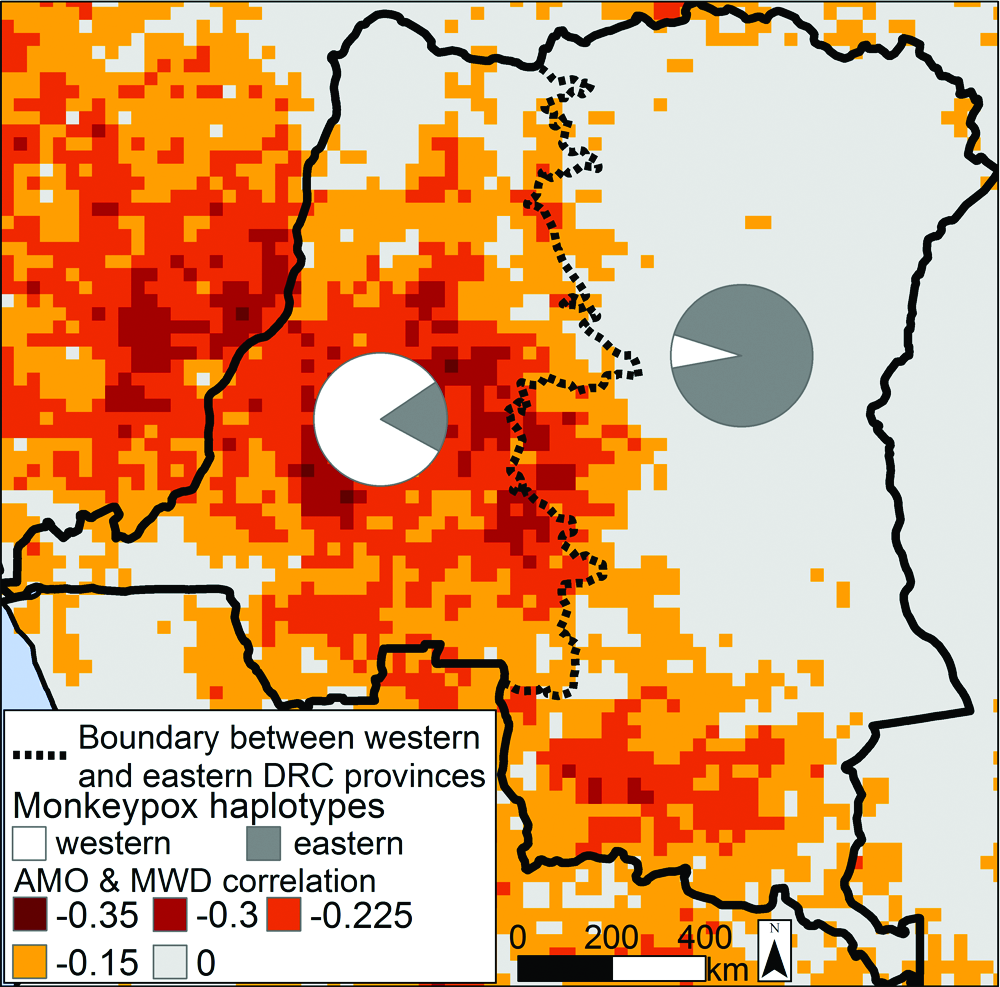

Supplement: Figure S7 — Association between climate and MPX genotypes in DRC at the national scale. MWD = maximum water deficit. AMO = Atlantic Multidecadal Oscillation. (TIF) [file pone.0066071.s007.tif]

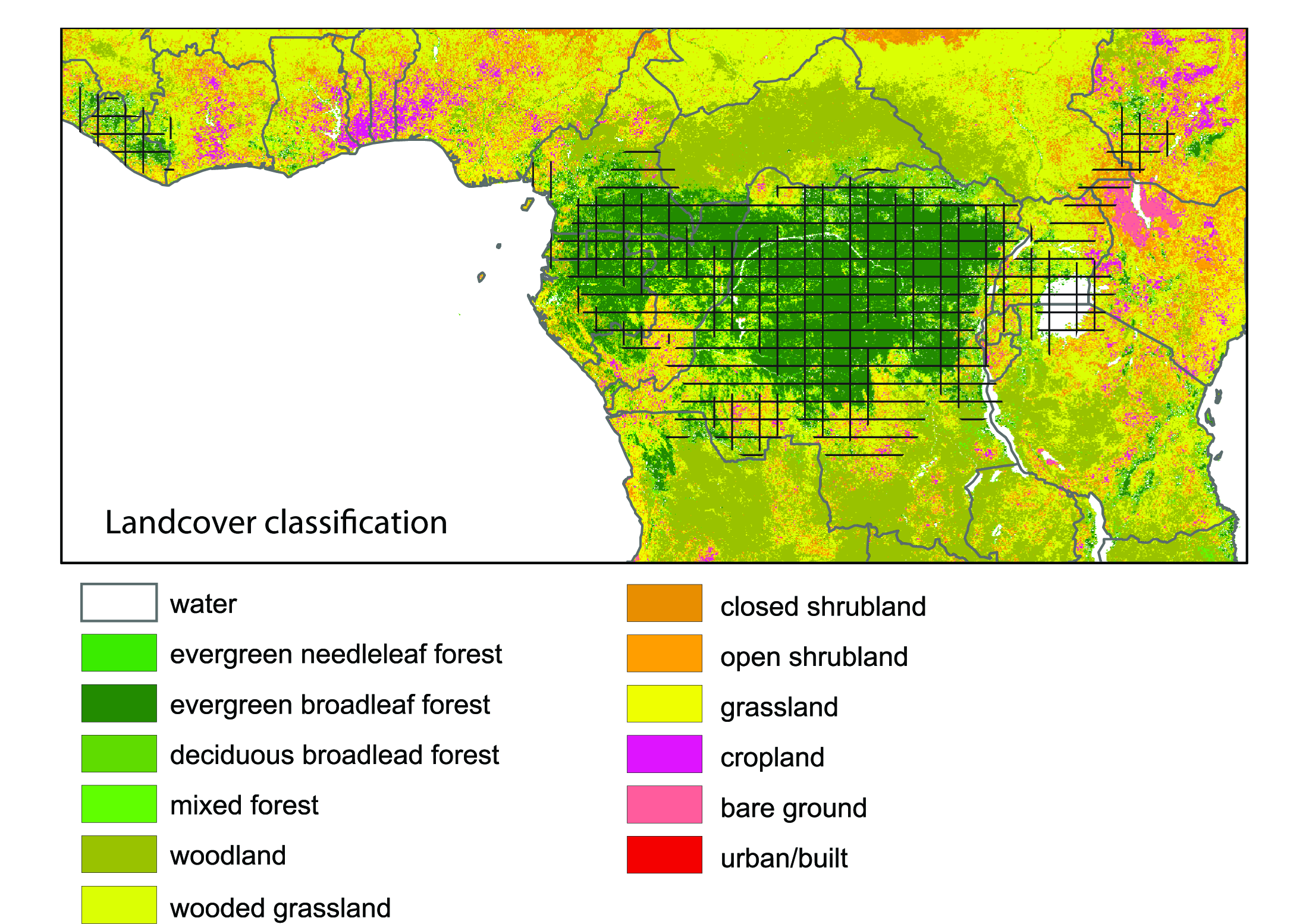

Supplement: Figure S8 — Land cover map of our study area. Hatched areas indicate a predicted increase in human MPX occurrence under the multi-model ensemble climate change scenarios for 2050–2060 (horizontal) and 2080–2090 (vertical). (TIF) [file pone.0066071.s008.tif]

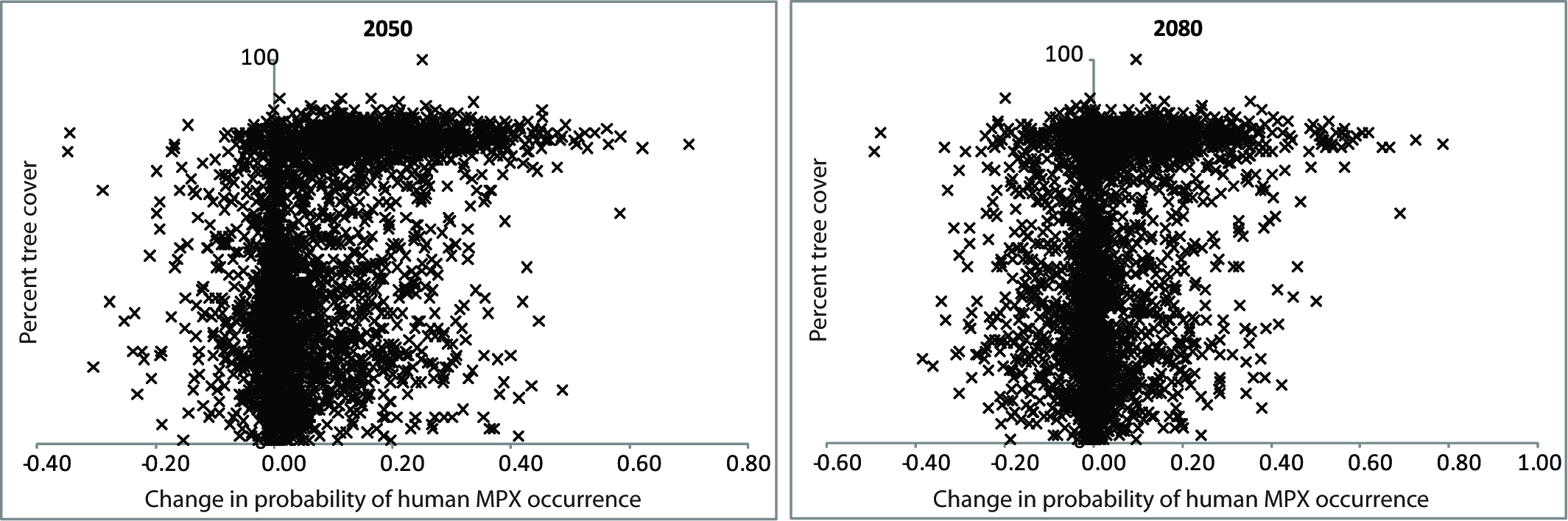

Supplement: Figure S9 — Plots of percent tree cover versus the predicted average increase in probability of human MPX occurrence for 2050 and 2080. Small increases in human MPX occurrence are predicted in both savanna and forest habitat types, but the largest increases are mainly located in forest areas. (TIF) [file pone.0066071.s009.tif]
