# Supplementary material for: Pathogen-Host Associations and Predicted Range Shifts of Human Monkeypox in Response to Climate Change in Central Africa
Source: PLoS One. 2013 Jul 31;8(7):e66071. doi: 10.1371/journal.pone.0066071 (PMC3729955; doi:10.1371/journal.pone.0066071)
Supplement: Figure S2 — Predictive models for MPXV reservoir species in Tropical Africa. Maxent predictions of MPXV reservoir species occurrence under contemporary climate conditions, using climate variables as predictor, and averages for eight climate change scenarios each for the periods 2050–2060 and 2080–2090. (PDF) [file pone.0066071.s002.pdf]

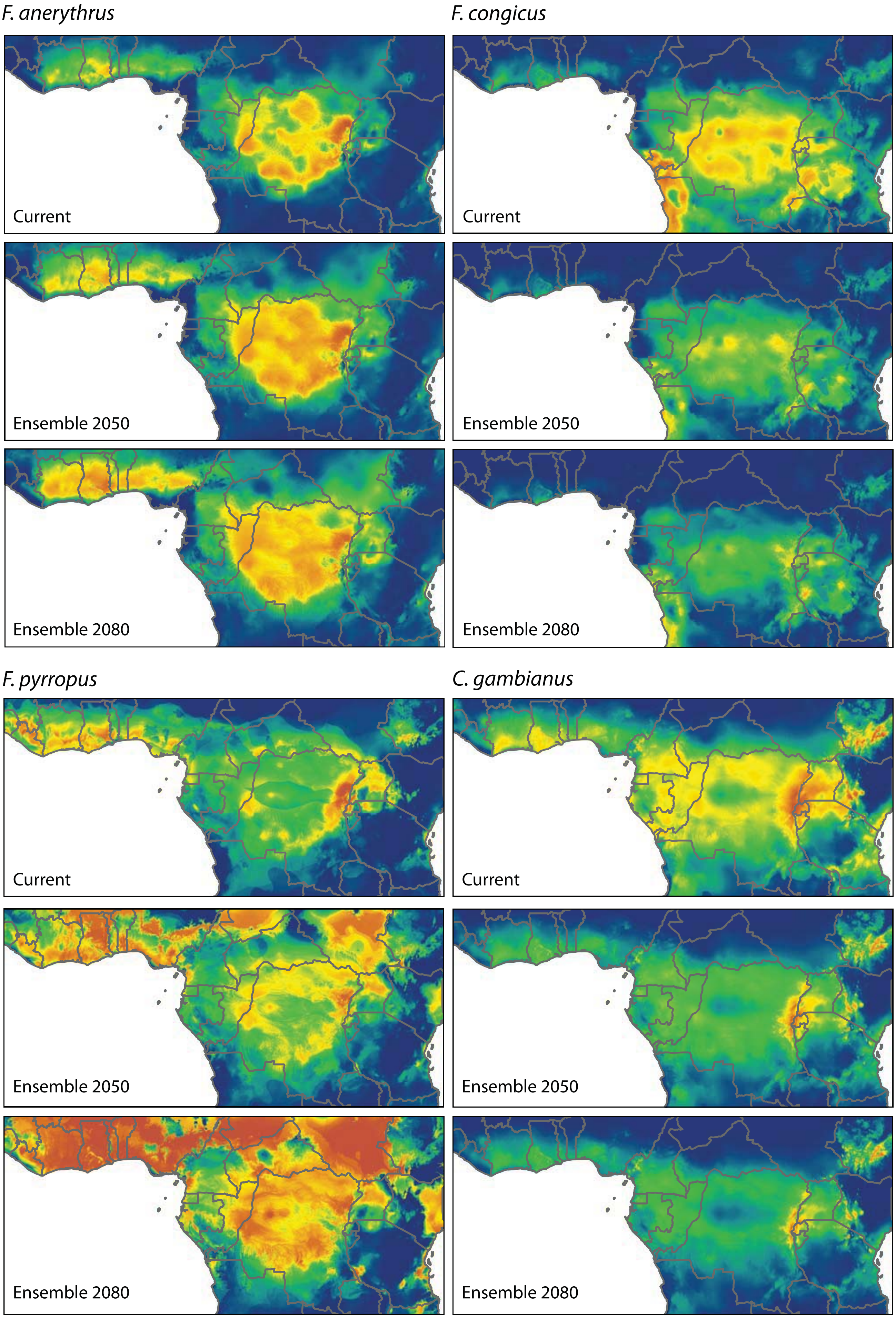

F. anerythrus
F. congicus
Current
Current
Ensemble 2050
Ensemble 2050
Ensemble 2080
Ensemble 2080
F. pyrropus
C. gambianus
Current
Current
Ensemble 2050
Ensemble 2050
Ensemble 2080
Ensemble 2080

A. africanus

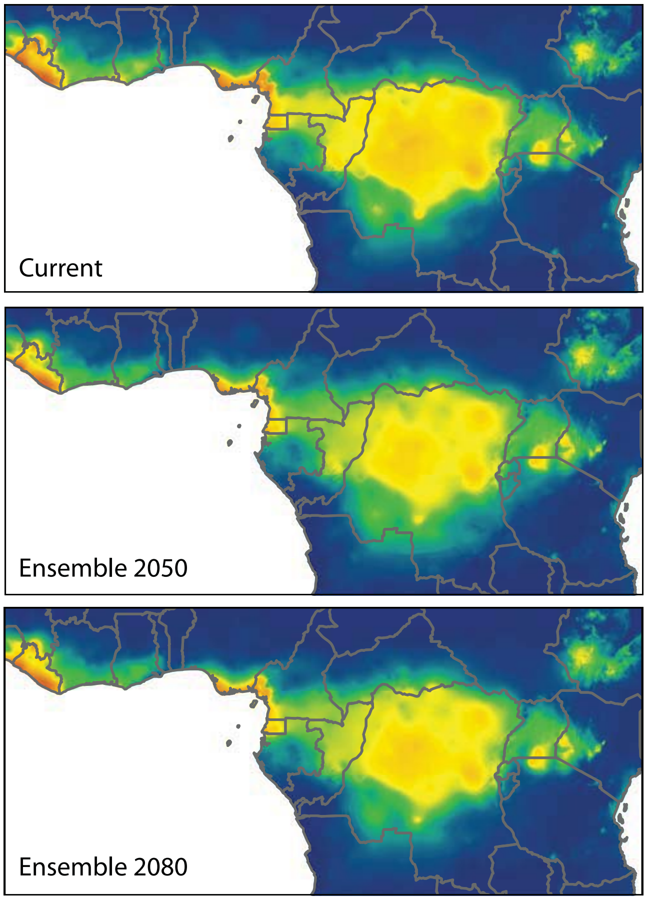

T. swinderianus

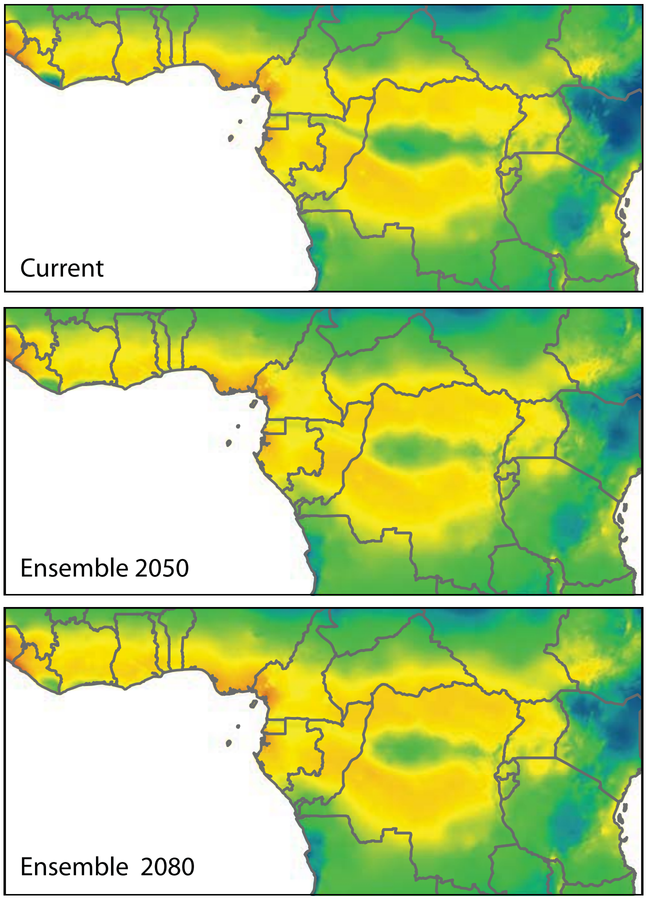

L. albigena

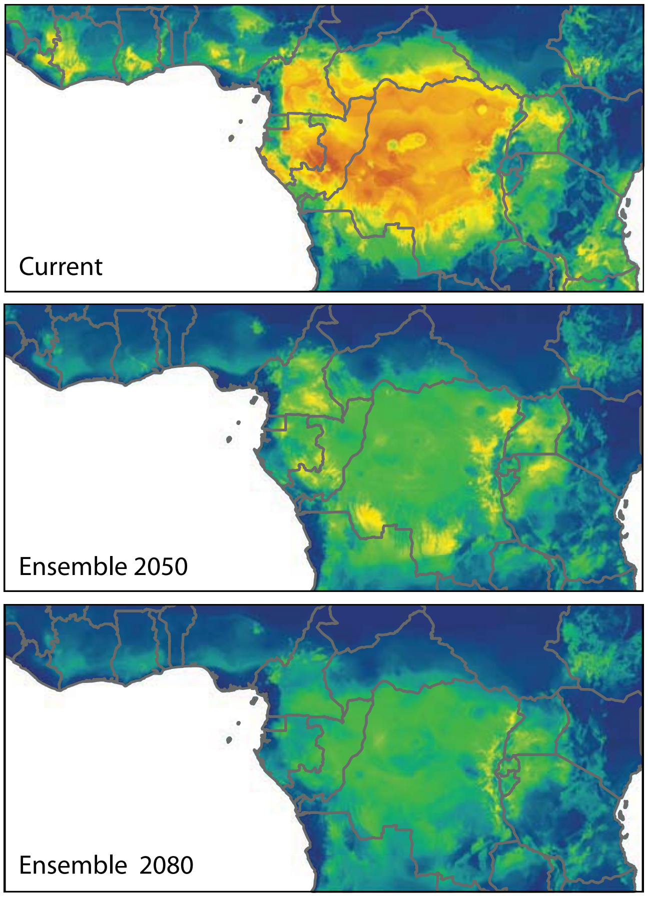

G. demidoff

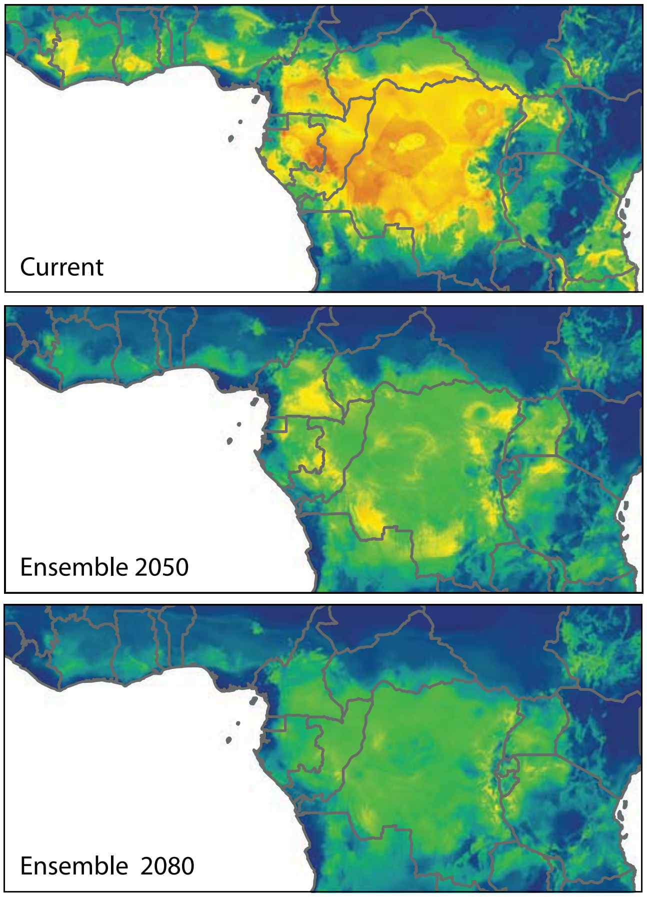

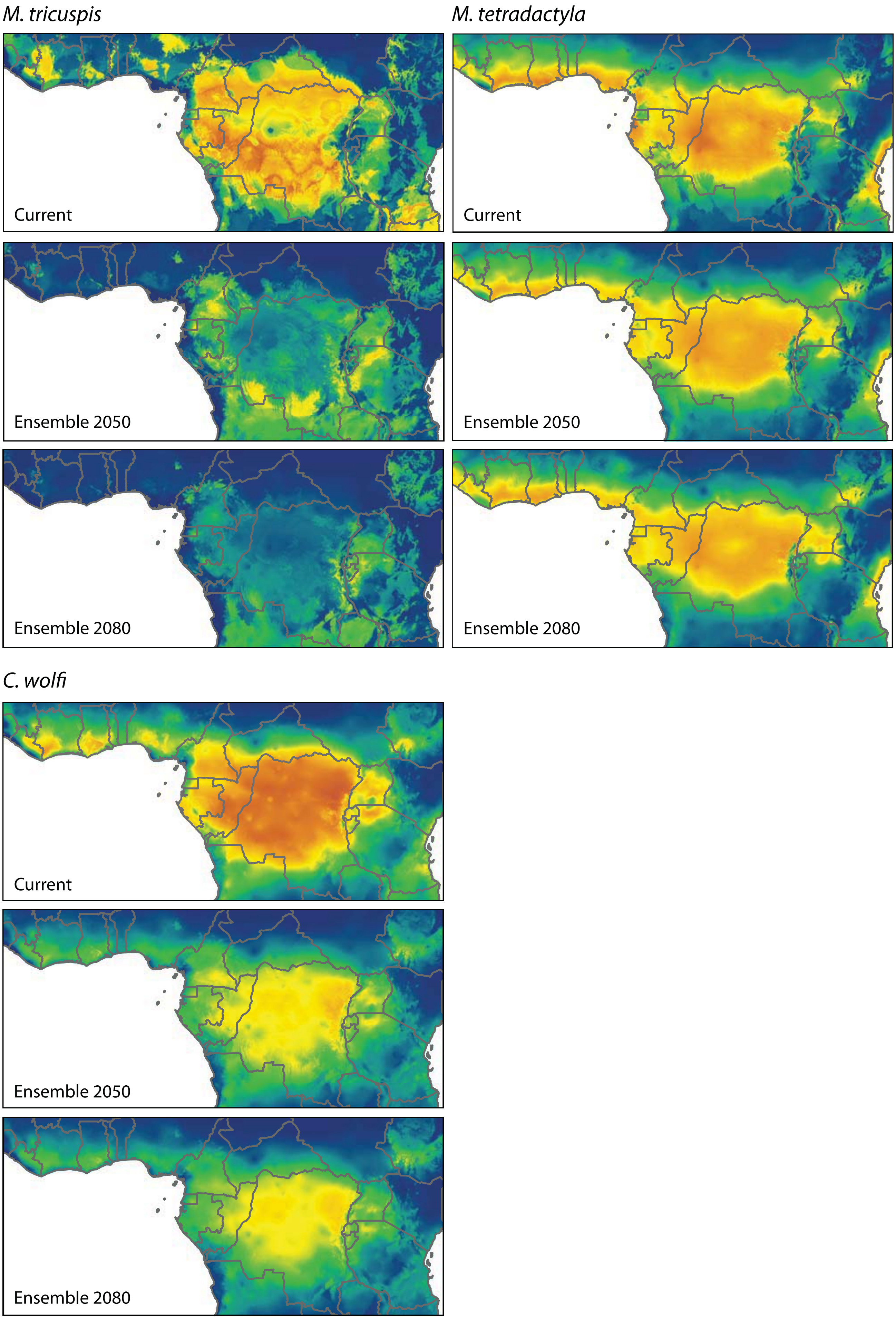

M. tricuspis
M. tetradactyla
Current
Current
Ensemble 2050
Ensemble 2050
Ensemble 2080
Ensemble 2080
C. wolfi
Current
Ensemble 2050
Ensemble 2080
