# Supplementary material for: Pathogen-Host Associations and Predicted Range Shifts of Human Monkeypox in Response to Climate Change in Central Africa
Source: PLoS One. 2013 Jul 31;8(7):e66071. doi: 10.1371/journal.pone.0066071 (PMC3729955; doi:10.1371/journal.pone.0066071)
Supplement: Table S1 — Intergovernmental Panel on Climate Change (IPCC) scenarios examined for Central Africa. (DOCX) [file pone.0066071.s010.docx]

**Table S1.** Intergovernmental Panel on Climate Change (IPCC) scenarios examined for Central Africa.

| **Year** | **IPCC Scenario** | **Global Climate Model** |
| --- | --- | --- |
| 2050 | A1b | CCCMA-CGCM31 |
|  |  | IPSL_CM4 |
|  | A2a | CCCMA-CGCM2 |
|  |  | CCCMA-CGCM3.1-T63 |
|  |  | HCCPR HADCM3 |
|  |  | MIROC3.2-HIRES |
|  | B2a | CCCMA-CGCM2 |
|  |  | CSIRO-MK2.0 |
| 2080 | A1b | CCCMA-CGCM31 |
|  |  | IPSL_CM4 |
|  |  | MPI_ECHAM5 |
|  |  | UKMO_HADCM3 |
|  | A2a | CCCMA-CGCM2 |
|  |  | HCCPR HADCM2 |
|  | B2a | CCCMA-CGCM2 |
|  |  | HCCPR HADCM2 |
