# Supplementary material for: Pathogen-Host Associations and Predicted Range Shifts of Human Monkeypox in Response to Climate Change in Central Africa
Source: PLoS One. 2013 Jul 31;8(7):e66071. doi: 10.1371/journal.pone.0066071 (PMC3729955; doi:10.1371/journal.pone.0066071)
Supplement: Text S1 — Estimation of Maximum Water Deficit, a measure of canopy moisture, in central DRC using remote sensing. (DOCX) [file pone.0066071.s011.docx]

**Supporting Information**

Text S1. Estimation of Maximum Water Deficit

To estimate drought in Sankuru’s forests, we first calculated total monthly precipitation at the 0.25º spatial resolution from 1998 to 2010 using the Tropical Rainfall Measuring Mission (TRMM), which is a satellite radar that has seen widespread use in mapping rainfall [[1](#_ENREF_1),[2](#_ENREF_2)]. Next, for each pixel (*i*,*j*) and each month *n* we calculated the water deficit (WD) defined as [[2](#_ENREF_2),[3](#_ENREF_3)]:

$${WD}_{n}\left( i,j \right)=\left\{ \begin{aligned} {WD}_{n-1}\left( i,j \right)-E\left( i,j \right)+P_{n}\left( i,j \right), &{WD}_{n-1}\left( i,j \right)+P_{n}\left( i,j \right)<E(i,j) \\ 0, &\text{otherwise} \end{aligned} \right.$$

where $P_{n}\left( i,j \right)$ denotes the monthly precipitation estimated from TRMM and $E(i,j)$ is the evapotranspiration constant. The maximum water deficit (MWD) for each pixel was defined as the maximum value of WD in each year. For the analysis reported here, the evapotranspiration constant was set to 100 mm month^-1^. This assumes that if evapotranspiration is less than 100, then forests in Sankuru experience drought. The assumption is supported for forests in the Amazon [[2](#_ENREF_2),[4](#_ENREF_4),[5](#_ENREF_5)], but we note that limited ground-based observations of evapotranspiration are available for other tropical forests. As a consequence, our predictions about drought in Sankuru could potentially be overestimated if forests in DRC are significantly more drought tolerant than Amazonian ones. Nevertheless, the evapotranspiration rate utilized here appears to be justified to the extent that it represents the best possible guess as to forest evapotranspiration rates in central DRC that is presently available, though we note that validating the estimate remains an important topic for future field campaigns.

Atlantic Multi-decadal Oscillation (AMO) index and water deficit correlation analysis

The Atlantic Multi-decadal Oscillation (AMO) is the oscillatory climate pattern of the North Atlantic Ocean, and correlated to air temperature and rainfall. The AMO signal is often determined from the patterns of sea surface temperature (SST) [6].

We examined the relationship between monthly water deficit anomalies and monthly AMO index using a spatio-temporal cross-correlation analysis. The monthly AMO index dataset for 1998-2010 was accessed through NOAA’s Earth System Research Laboratory (<http://www.esrl.noaa.gov/psd/data/timeseries/AMO/> Accessed February 2011), providing users with a monthly time series of the AMO index that is calculated from the Kaplan SST dataset [6].

Monthly water deficits (WD) were calculated using TRMM monthly rainfall measurements of 1998-2010 (see above). Monthly water deficit anomalies were then calculated for each year (y) on a pixel-by-pixel (i,j) basis as a departure from the 1998-2010 mean (**WD1998-2010**), excluding the measurement from year (y) and normalized by the standard deviation STD:

$$WD_{y_{anomaly}}(i,j)=\frac{WD_{y}(i,j)-<WD_{1998-2010}(i,j)>}{STD(WD_{1998-2010}(i,j))}$$

Pearson’s correlation coefficient between the ranks of variables was calculated on pixel-to-pixel basis using:

where r is known as the Spearman rho between ranked variables xi and yi at a pixel location, with $\bar{x}$and $\bar{y}$being the average of variables over the time interval. In this case, xi and yi are monthly water deficit anomalies and monthly AMO index respectively over the period 1998-2010.

1. Kummerow C, Barnes W, Kozu T, Shiue J, Simpson J (1998) The Tropical Rainfall Measuring Mission (TRMM) sensor package. Journal of Atmospheric and Oceanic Technology 15: 809-817.

2. Asefi-Najafabady S, Saatchi S (accepted pending revisions) Impacts of climate variability on African Tropical Rainforest. Geophysical Research Letters.

3. Aragao L, Malhi Y, Roman-Cuesta RM, Saatchi S, Anderson LO, et al. (2007) Spatial patterns and fire response of recent Amazonian droughts. Geophysical Research Letters 34.

4. da Rocha HR, Goulden ML, Miller SD, Menton MC, Pinto L, et al. (2004) Seasonality of water and heat fluxes over a tropical forest in eastern Amazonia. Ecological Applications 14: S22-S32.

5. Shuttleworth WJ (1989) Micrometeorology of temperate and tropical forest. Philosophical Transactions of the Royal Society of London Series B-Biological Sciences 324: 299-334.

6. Enfield DB, Mestas-Nunez AM, and Trimble PJ (2001) The Atlantic Multidecadal Oscillation and its relationship to rainfall and river flows in the continental U.S. Geophysical Research Letters 28: 2077-2080
