# Supplementary material for: Pathogen-Host Associations and Predicted Range Shifts of Human Monkeypox in Response to Climate Change in Central Africa
Source: PLoS One. 2013 Jul 31;8(7):e66071. doi: 10.1371/journal.pone.0066071 (PMC3729955; doi:10.1371/journal.pone.0066071)
Supplement: Text S2 — Spatial autocorrelation and spatial dependence. (DOCX) [file pone.0066071.s012.docx]

Text S2. Spatial autocorrelation and spatial dependence

Spatial clustering of sites where human MPX cases have been observed may result in spatial autocorrelation. There are at least four factors that might cause spatial autocorrelation: 1) sampling bias; 2) differential immune responses of reservoir and human populations in different locations; 3) cultural differences in human behavior affecting MPXV transmission potential; and 4) dependence of virus or host species on environmental factors that are inherently spatially autocorrelated (often termed spatial dependence). Spatial autocorrelation in niche modeling may result in inaccurate distribution models and artificially inflated measures of model performance [e.g., 1,2], and it is sometimes suggested that it should be corrected for prior to model fitting. However, it is often difficult to distinguish the possible causes of spatial autocorrelation, yet their distinction is crucial in making inferences regarding disease distribution and relevance of environmental factors. In particular, spatial dependence of virus or host occurrence on environmental factors likely results in spatial autocorrelation, yet is one of the properties we are most interested in when modeling disease distributions. Moreover, differential but spatially correlated immune responses in hosts may similarly be related to environmental heterogeneity, for instance due to past selection pressures on the host’s genome, or due to cross-immunizing pathogens that circulate in particular regions. Removal of spatial autocorrelation from the input dataset may result in the elimination of relevant information regarding the relationship between disease occurrence and environmental factors. In our dataset of human MPX cases, sampling bias towards the Sankuru district may be an underlying factor of spatial autocorrelation. To reduce the effect of this potential bias, we imposed a minimum distance among sampling sites of 10 km. Thus, potential factors that could result in spatial autocorrelation in our final dataset include regional differences in human behavior, differential immune responses, and spatial dependence of virus or hosts on the environment. To further assess the potential impact of spatial autocorrelation on model performance, instead of *a priori* removing spatial autocorrelation, we used spatially independent training and test datasets.

References

1. Parolo G, Rossi G, Ferrarini A (2008) Toward improved species niche modelling: Arnica montana in the Alps as a case study. Journal of Applied Ecology 45: 1410-1418.

2. Veloz SD (2009) Spatially autocorrelated sampling falsely inflates measures of accuracy for presence-only niche models. Journal of Biogeography 36: 2290-2299.
